# Efficacy of non-invasive brain stimulation for post-stroke sleep disorders: a systematic review and meta-analysis

**DOI:** 10.3389/fneur.2024.1420363

**Published:** 2024-10-30

**Authors:** Linyu Huang, Xingling Zhang, Jie Zhang, Long Li, Xianyu Zhou, Tingyu Yang, Xuemei An

**Affiliations:** ^1^School of Nursing, Chengdu University of Traditional Chinese Medicine, Chengdu, China; ^2^Nursing Department, Hospital of Chengdu University of Traditional Chinese Medicine, Chengdu, China; ^3^Administrative Management Department, Deyang Hospital of Chengdu University of Traditional Chinese Medicine, Deyang, China

**Keywords:** non-invasive brain stimulation, transcranial magnetic stimulation, transcranial direct current stimulation, stroke, sleep disorder, insomnia, meta-analysis

## Abstract

**Objective:**

This study aimed to systematically assess the clinical efficacy of non-invasive brain stimulation (NIBS) for treating post-stroke sleep disorders (PSSD).

**Methods:**

We conducted thorough literature search across multiple databases, including PubMed, Web of Science, EmBase, Cochrane Library, Scopus, China Biology Medicine (CBM); China National Knowledge Infrastructure (CNKI); Technology Periodical Database (VIP), and Wanfang Database, focusing on RCTs examining NIBS for PSSD. Meta-analyses were performed using RevMan 5.4 and Stata 14.

**Results:**

Eighteen articles were reviewed, including 16 on repetitive Transcranial Magnetic Stimulation (rTMS), one on Theta Burst Stimulation (TBS), and two on transcranial Direct Current Stimulation (tDCS). Meta-analysis results indicated that rTMS within NIBS significantly improved the Pittsburgh Sleep Quality Index (PSQI) score (MD = −1.85, 95% CI [−2.99, −0.71], *p* < 0.05), the 17-item Hamilton Depression Rating Scale (HAMD-17) score [MD = −2.85, 95% CI (−3.40, −2.30), *p* < 0.05], and serum brain-derived neurotrophic factor (BDNF) levels [MD = 4.19, 95% CI (2.70, 5.69), *p* < 0.05], while reducing the incidence of adverse reactions [RR = 0.36, 95% CI (0.23, 0.55), *p* < 0.05]. TBS significantly improved the PSQI score in patients with PSSD (*p* < 0.05). Conversely, tDCS significantly improved the HAMD-17 score in PSSD patients [MD = −1.52, 95% CI (−3.41, −0.64), *p* < 0.05]. Additionally, rTMS improved sleep parameters, including Stage 2 sleep (S2%) and combined Stage 3 and 4 sleep (S3 + S4%) (*p* < 0.05), while tDCS improved total sleep time (TST) and sleep efficiency (SE) (*p* < 0.05).Subgroup analysis results indicated: (1) Both LF-rTMS and HF-rTMS improved PSQI scores (*p* < 0.05). (2) Both rTMS combined with medication and rTMS alone improved PSQI scores (*p* < 0.05). Compared to the sham/blank group, the rTMS group showed improvements in SE, sleep latency (SL), S1%, S3 + S4%, and REM sleep (REM%). The rTMS combined with medication group showed improved SL compared to the medication-only group (*p* < 0.05).

**Conclusion:**

NIBS effectively improves sleep quality, structure, depression levels, and BDNF levels in PSSD patients, while also being safe. Further investigations into the potential of NIBS in PSSD treatment may provide valuable insights for clinical applications.

**Systematic review registration:**

https://www.crd.york.ac.uk/prospero/, CRD42023485317.

## 1 Introduction

Post-stroke Sleep Disorders (PSSD) are prevalent complications following a stroke, affecting up to 78% of patients (1), with rates of post-stroke insomnia (PSI) ranging from 30.1 to 46.5% (2–4). PSSD refer to a group of clinical syndromes that either persist or worsen following a stroke and meet the diagnostic criteria for sleep disorders. These syndromes include insomnia, sleep-disordered breathing (SDB), excessive daytime sleepiness (EDS), and restless legs syndrome (RLS) (5). PSSD is primarily characterized by a reduction in total sleep time, prolonged sleep onset latency, increased frequency of awakenings, extended duration of light sleep, and a decrease in the amount of rapid eye movement (REM) sleep. Research indicates that PSSD severely impact patients’ quality of life, mental health, and motor function recovery. These effects contribute to exacerbated neurological damage and delayed neural regeneration, ultimately hindering the overall recovery process (6–8). Persistent sleep disorders may even precipitate a secondary stroke, heightening the risk of recurrence, disability, and mortality (5).

There is growing recognition that sleep disturbances significantly affect neural plasticity, a process fundamental to post-stroke recovery. Neural plasticity refers to the brain’s ability to reorganize itself in response to injury, and sleep plays a crucial role in facilitating synaptic plasticity and neural circuit optimization (9). Sleep plays a crucial role in memory consolidation, cognitive function, and the homeostatic optimization of neural circuits, allowing for the replay-based consolidation of specific neural circuits essential for maintaining optimal brain function (10). Sleep disruptions, especially following a stroke, impair these plasticity-related processes, potentially delaying functional recovery. Additionally, sleep is essential for processes like memory consolidation and neural homeostasis, which are integral to maintaining cognitive and motor function. These findings highlight the interplay between sleep and plasticity in the recovery of patients with cerebrovascular diseases (10, 11).

Despite their significant effects on prognosis and quality of life, sleep disorders often remain under-recognized and inadequately managed, leading to poorer recovery outcomes (11). Therefore, addressing PSSD with appropriate therapeutic strategies is crucial. Currently, treatments involve pharmacological and non-pharmacological approaches. Pharmacological interventions typically include benzodiazepines (e.g., lorazepam), non-benzodiazepines (e.g., zolpidem), melatonin receptor agonists (e.g., ramelteon), and sedative antidepressants. However, long-term use of these medications can lead to various side effects, drug dependence, and withdrawal reactions. Therefore, it is crucial to identify safe, effective, and short-duration non-pharmacological treatments for PSSD patients. Non-pharmacological treatments include cognitive behavioral therapy, light therapy, acupuncture, traditional Chinese medicine, and non-invasive brain stimulation (12–14). Although cognitive behavioral therapy (CBT) for insomnia is the preferred first-line non-pharmacologic treatment and has proven effectiveness (15), its widespread use is limited by a shortage of therapists and economic constraints. While the Internet offers cost-effective promotion possibilities, standardization and personalization of digital therapies still need improvement. Additionally, both acupuncture-moxibustion and traditional Chinese medicine have limitations, with their efficacy potentially varying due to the technical skill of practitioners (16, 17).

Non-invasive brain stimulation (NIBS) has garnered interest as a promising treatment for PSSD, particularly for its potential to modulate cortical excitability and enhance neuroplasticity, which are crucial for functional recovery. Emerging evidence suggests that NIBS, by targeting neural circuits, can induce synaptic plasticity, reorganize neural networks, and optimize sleep architecture, making it a valuable tool in the treatment of PSSD (18). Studies have shown that NIBS can modulate cortical excitability, with techniques like transcranial magnetic stimulation (TMS) and transcranial direct current stimulation (tDCS) effectively reducing cortical excitability, increasing slow-wave sleep duration, and improving overall sleep quality in stroke patients (19).

NIBS primarily consists of transcranial electrical stimulation (TES) and transcranial magnetic stimulation (TMS) (20). TMS stimulation modes primarily includes repetitive TMS (rTMS) and theta burst stimulation (TBS). TMS utilizes Faraday’s law of electromagnetic induction, where a magnetic coil generates a pulsed magnetic field that penetrates the scalp and skull without attenuation, reaching the cerebral cortex. TMS can transiently modulate the excitability and plasticity of the target brain region, influence the excitability of cortical neurons, and promote neurotransmitter release, thereby improving sleep disorders. Compared to cognitive-behavioral interventions and pharmacotherapy, TMS optimizes sleep architecture, modulate sleep quality, and supports both clinical compliance and long-term therapeutic outcomes (18). Repetitive transcranial magnetic stimulation (rTMS) employs electromagnetic induction to non-invasively stimulate the cerebral cortex, modulating the excitability of the stimulation site and its connected brain regions. High-frequency rTMS can increase cortical excitability, whereas low-frequency rTMS can inhibit neuronal excitability and induce long-term potentiation (LTP) and long-term depression (LTD) (21). Studies suggest that rTMS may regulate the sleep–wake system by reducing cortical excitability, modulating neurotransmitter activity (such as GABA, 5-HT, and BDNF) and increasing cerebral blood flow, thereby improving sleep quality in stroke patients with sleep disorders (22, 23). Moreover, magnetic field exposure may influence melatonin synthesis and secretion in the pineal gland, as well as regulate neurotransmitter levels, including 5-HT, norepinephrine (NA), and acetylcholine (ACh), all of which are essential in maintaining a normal sleep–wake cycle and overall physiological function (24). TBS, a type of TMS, has a frequency similar to the brain’s hippocampal theta wave, closely mirroring the physiological state of neurophysiological activity, and is believed to induce NMDA receptor-dependent LTP and LTD by mimicking the brain’s theta rhythm (21, 25). With brief stimulation sessions lasting 40 to 190 s, TBS can induce prolonged changes in cortical excitability that persist for 20 to 30 min after stimulation, further supporting its role in regulating synaptic plasticity and cortical excitability in the treatment of post-stroke insomnia (25).

TES stimulation modes primarily includes transcranial direct current stimulation (tDCS), transcranial alternating current stimulation (tACS), and transcranial random noise stimulation (tRNS) (26). TES modulates brain activity by applying electrical stimulation to specific scalp areas. tDCS applies a sustained weak current to specific brain regions, altering neuronal membrane potential and modulating excitability (27). Anodal tDCS typically depolarizes the membrane, enhancing cortical excitability, while cathodal tDCS hyperpolarizes it, reducing excitability. Numerous studies have demonstrated the effectiveness of tDCS in treating patients with sleep disorders; however, the exact neurophysiological mechanisms by which tDCS influences neuronal activity remain unclear. Currently, the academic community widely accepts two possible mechanisms (28). The first is the direct effect, where the anodal electrode increases the excitability of the corresponding cortical area by depolarizing the resting membrane potential of neurons, while the cathodal electrode reduces excitability by hyperpolarizing it (29). The second is the delayed effect, in which tDCS may induce neuronal remodeling by modulating synaptic transmission, relying on mechanisms similar to LTP and LTD (30). Additionally, research indicates that tDCS can influence neurotransmitter levels, such as *γ*-aminobutyric acid (GABA) and glutamate, further modulating functional connectivity and synaptic plasticity between neurons (31, 32). Conversely, tACS regulates endogenous neural oscillations by altering the synchronization or desynchrony of neural activities, leading to neural oscillation entrainment or resonance and enhancing neural plasticity (33, 34).

Recent studies have demonstrated the efficacy of NIBS in enhancing sleep quality in post-stroke patients, with improvements in sleep latency, total sleep time, and polysomnography indicators (35, 36). The emerging role of NIBS in modulating neural circuits “*in vivo*” is particularly noteworthy, as it offers a new avenue for addressing the complex interactions between sleep, plasticity, and stroke recovery. However, despite promising results, current research on NIBS is still limited by small sample sizes, varying inclusion criteria, and heterogeneous methodologies, leading to inconclusive evidence regarding its therapeutic efficacy and safety. Consequently, this study aims to systematically evaluate the clinical efficacy of NIBS in treating post-stroke sleep disorders, with a focus on its impact on neural plasticity and functional recovery. By addressing these gaps, we hope to provide robust evidence to guide clinical decision-making and optimize therapeutic strategies for patients with.

## 2 Materials and methods

The meta analysis was registered with PROSPERO No. CRD42023485317 and complied with the PRISMA statement (37).

### 2.1 Search strategy

A systematic search for randomized controlled trials (RCTs) evaluating the efficacy of NIBS in treating PSSD was conducted in databases including PubMed, Web of Science, EmBase, Cochrane Library, Scopus, China Biology Medicine(CBM), China National Knowledge Infrastructure(CNKI), Technology Periodical Database(VIP) and Wanfang Database. The search covered the period from each database’s inception to November 2023, using a combination of MeSH terms and entry terms. Two researchers (ZXL and ZJ) independently conducted the search following a consensus on the search strategy. The detailed search strategy is shown in Supplementary material.

### 2.2 Eligibility criteria

The following were among the study’s inclusion criteria: this study is a RCT examining the efficacy of NIBS in treating PSSD. The participants comprised adults over 18 years old, with a confirmed diagnosis of PSSD, meeting the established criteria for both stroke and sleep disorders, irrespective of gender or race. The intervention involved administering NIBS to the experimental group, while the control group received routine treatment combined with pharmacotherapy or sham stimulation. The primary outcome measure was sleep quality, assessed using the Pittsburgh Sleep Quality Index (PSQI) or Polysomnography (PSG). Secondary outcomes included the Hamilton Depression Rating Scale (HAMD), brain-derived neurotrophic factor (BDNF) levels, and the incidence of adverse events. Excluded literature includes those with non-comparable baselines or poor study designs, incomplete data, unavailability of raw data and full text, conference abstracts, animal experiments, trial protocols, case reports, expert consensus, guidelines, meta-analyses, reviews, and duplicate publications.

### 2.3 Literature screening and data extraction

Two researchers (ZXL and ZJ) independently screened the literature using EndNote X9 software, following the predefined inclusion and exclusion criteria, and initially reviewed titles and abstracts for duplicates before examining the full texts. In cases of literature with incomplete data, attempts were made to contact the original authors for additional information. Literature for which data remained inaccessible after exhaustive efforts was excluded. This screening process was cross-validated by the two researchers, with any disputes resolved by a third party. The data extracted from the selected studies included author, publication year, study population, sample size, participant age, NIBS intervention parameters (such as frequency, location, intensity, and duration), and outcome measures. The timing of post-intervention assessment was standardized across studies to harmonize the treatment evaluation criteria.

### 2.4 Quality assessment

Two researchers (ZXL and ZJ) assessed the quality of the included studies using the Risk of Bias (RoB) tool recommended by the Cochrane Collaboration, which evaluates six domains: selection bias, performance bias, detection bias, attrition bias, reporting bias, and other biases. Each domain’s bias risk was categorized as “high risk,” “low risk,” or “unclear.” Discrepancies were resolved through discussion with a third researcher.

### 2.5 Statistical analysis

Meta-analysis was performed using RevMan 5.4 software. The outcome measures of the included studies were synthesized using weighted mean difference (MD) for continuous outcomes and relative risk (RR) for binary outcomes, each with a 95% confidence interval (95%CI) and a significance level of *α* = 0.05. Heterogeneity was assessed using the Cochrane Q test and *I^2^* statistic. A fixed effect model was employed for meta-analysis when heterogeneity was not significant (Q test *p* > 0.1, *I^2^* < 50%). Conversely, significant heterogeneity (Q test *p* ≤ 0.1, *I^2^* ≥ 50%) necessitated the use of a random effects model. Additionally, Stata 14 software facilitated bias assessment, sensitivity analyses, and subgroup analyses to identify heterogeneity sources.

## 3 Results

### 3.1 Selection of the results and study characteristics

**Figure 1:**
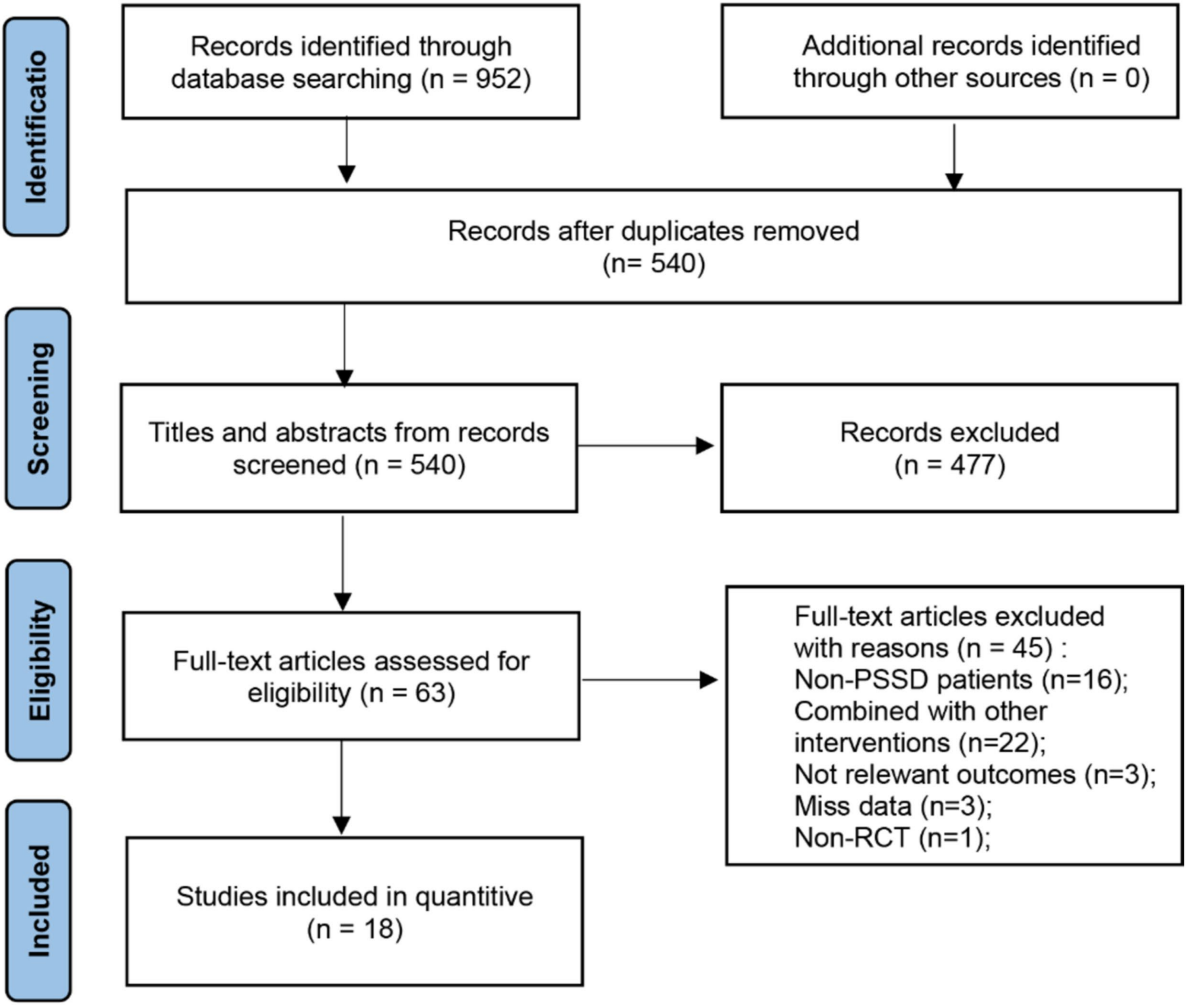
PRISMA flow chart on selection and inclusion of studies.

A total of 952 articles were initially retrieved, with 412 duplicates removed, resulting in a final meta-analysis that included 18 studies comprising 1,482 patients. The detailed screening procedure is presented in Figure 1. Among the included studies, one was a multi-arm trial (36), two examined tDCS (32, 38), one focused on TBS (36), and 16 investigated rTMS (36, 39–52, 82). The rTMS studies were divided into low frequency stimulation (LF-rTMS, ≤1 Hz) and high frequency stimulation (HF-rTMS, ≥5 Hz). Three studies (43, 46, 48) utilized HF-rTMS, while LF-rTMS was employed in 11, with 1 Hz and 10 Hz being the most common frequencies. One study (45) did not specify the stimulation frequency, and another (41) alternated between 1 Hz and 11 Hz. Stimulation areas commonly targeted the dorsolateral prefrontal cortex (DLPFC), including left (l-DLPFC), right (r-DLPFC), or bilateral (b-DLPFC) regions. Most studies used figure-of-eight coils for stimulation, and stimulus intensities ranged from 80 to 120% of the resting motor threshold (RMT). Seven studies (39–41, 47, 49, 50, 82) compared NIBS with pharmacotherapy in experimental and control groups, another seven (38, 42, 43, 45, 46, 48, 51) compared combined NIBS and pharmacotherapy against pharmacotherapy alone, and four (32, 36, 44, 52) evaluated NIBS against sham or no intervention. Common pharmacological agents used in combination with NIBS included alprazolam, escitalopram, and zolpidem, as detailed in Table 1.

**Table 1:** Characteristics of included studies.

Study	Sample size(E/C)	Age (E/C, years)	Type of stroke	Interventions		Stimulation frequency (Hz)	stimulated areas	stimulated coil	Stimulus intensity	Duration	Outcome indicators
				C	E						
Luo et al. (39)	55/55	62.73 ± 6.07/61.24 ± 5.42	IS and HS	alprazolam tablet	rTMS	1 Hz	−	−	500Gs	30 min/d, 4wks	PSQI、Adverse reactions
Chen et al. (40)	32/32	64.2 ± 6.9/66.5 ± 6.9	IS and HS	Alprazolam	rTMS	1 Hz	FC	−	500Gs	30 min/d, 6d/wk., 4wks	PSQI
Sheng et al. (41)	50/48	61.17 ± 12.56/57.24 ± 11.1	IS and HS	Dexzopiclone Tablets	rTMS	1mHz 20 min after 11mHz 5 min	PCZ	−	500Gs	20 min/d, 5d/wk., 2wks	PSQI、PSG
Zhu et al. (42)	30/30	65.97 ± 10.51/65.90 ± 9.50	IS	Zolpidem Tartrate Tablets+false stimulus	Zolpidem Tartrate Tablets+ILF-TMS	0.2 Hz	−	Circular coil	500GS	20 min/d, 10ds	PSQI、PSG
Chen et al. (43)	32/31	64.06 ± 6.82/65.16 ± 9.18	IS and HS	Escitalopram +false stimulus	Escitalopram +rTMS	10 Hz	l-DLPFC	Figure-of-eight coil	90%MT	1session/d, 10 consecutive treatment days. 4wks	PSQI、PSG、HAMD-17
Ding et al. (44)	46/46	70 ± 4/72 ± 4	IS and HS	false stimulus	rTMS	1 Hz	b-DLFC	−	80%MT	1session/d, 2wks	PSQI、PSG、BDNF、 Adverse reactions
Armalia et al. (45)	24/24	18–65	IS	Medication (not specified)	Medication (not specified) + rTMS	−	−	−	−	−	PSQI
Chen and Fu (46)	45/45	70 ± 5/70 ± 5	IS and HS	Bailemian capsules+false stimulus	Bailemian capsules+rTMS	10 Hz	b-DLFC	Figure-of-eight coil	80%RMT	1session/d, 5ds/wk., 4wks	PSQI、BDNF、Adverse reactions
Xu et al. (47)	30/28	65.7 ± 6.1/64.2 ± 5.9	IS and HS	alprazolam tablets	rTMS	1 Hz	r-DLFC	Figure-of-eight coil	500Gs	20 min/d, 1mo	PSQI、Adverse reactions
Dong (48)	43/43	60.96 ± 5.13/61.32 ± 4.67	IS and HS	Escitalopram oxalate tablets+false stimulus	Escitalopram oxalate tablets+rTMS	10 Hz	−	Figure-of-eight coil	80%MT	20 min/d, 5ds/wk., 4wks	PSQI、HAMD-17、BDNF
Gu et al. (32)	22/22	54.2 ± 12.66/58.6 ± 12.58	IS and HS	false stimulus	tDCS	2 mA	Anode:l-DLPFC Cathode:r-DLPFC	Bipolar Electrode Pads	2 mA	20 min/d, 5ds/wk., 4wks	PSQI、PSG、HAMD-17
Han (38)	44/43	53.68 ± 7.52/55.23 ± 7.79	IS and HS	Fluoxetine Hydrochloride Dispersible Tablets	Fluoxetine Hydrochloride Dispersible Tablets+HD-tDCS	2 mA	l-DLPEC	High definition circular electrode	−	1session/d, 4wks	PSQI、HAMD-17
Huang (49)	45/45	61.06 ± 4.65/61.20 ± 4.69	IS	Dexzopiclone tablets	rTMS	1 Hz	r-DLPFC	Figure-of-eight coil	−	20 min/d, 14ds	PSQI
Qi et al. (50)	46/45	63.12 ± 6.07/63.75 ± 5.92	IS and HS	Alprazolam	rTMS	1 Hz	r-DLFC	Figure-of-eight coil	80% ~ 120%MT	20 min/d, rest 2ds after 5ds of treatment, 1mo	PSQI、Adverse reactions
Xiao et al. (51)	30/30	−	IS	Estazolam +false stimulus	Estazolam +rTMS	1 Hz	b-DLPFC and POR	Figure-of-eight coil	120%MT	30 min/d, 2wks	PSG、BDNF、Adverse reactions
Zhang (36)	13/13	59.33 ± 8.25/59.50 ± 11.18	IS and HS	Routine rehabilitation	Routine rehabilitation+TBS	3 pulses/clump, inter-clump: 5 Hz, intra-clump: 50 Hz	r-DLPFC	Figure-of-eight coil	70%RMT	1session/d, 7ds/wk., 2wks	PSQI、HAMD
Zhang (36)	13/13	63.75 ± 9.11/59.50 ± 11.18	IS and HS	Routine rehabilitation	Routine rehabilitation+rTMS	1 Hz	r-DLPFC	Figure-of-eight coil	80%RMT	25 min/d, 7ds/wk., 2wks	PSQI, HAMD, Adverse reactions
Zhong et al. (52)	50/50	63.86 ± 8.78/62.88 ± 7.99	IS	Routine rehabilitation	Routine rehabilitation+rTMS	1 Hz	−	Circular coil	−	20 min/d, 7ds	PSQI
An et al. (82)	42/45	60 ± 8/61 ± 8	IS	Estazolam	rTMS	1 Hz	r-DLFC	Figure-of-eight coil	80% ~ 120%MT	1session/d, 4wks	PSQI、PSG、Adverse reactions

Stimulation sites are denoted as follows: DLPFC (dorsolateral prefrontal cortex), b-DLPFC (bilateral dorsolateral prefrontal cortex), l-DLPFC (left dorsolateral prefrontal cortex), r-DLPFC (right dorsolateral prefrontal cortex), POR (parietal-occipital region), and r-DLFC (right dorsolateral frontal cortex). The frontal cortex is abbreviated as FC, and b-DLFC represents the bilateral dorsolateral frontal cortex. PCZ is defined as 1 cm posterior to the parietal CZ area. Stimulus intensity is indicated by MT (motor threshold), RMT (resting motor threshold), and GS (Gauss).

### 3.2 Quality assessment

**Figure 2:**
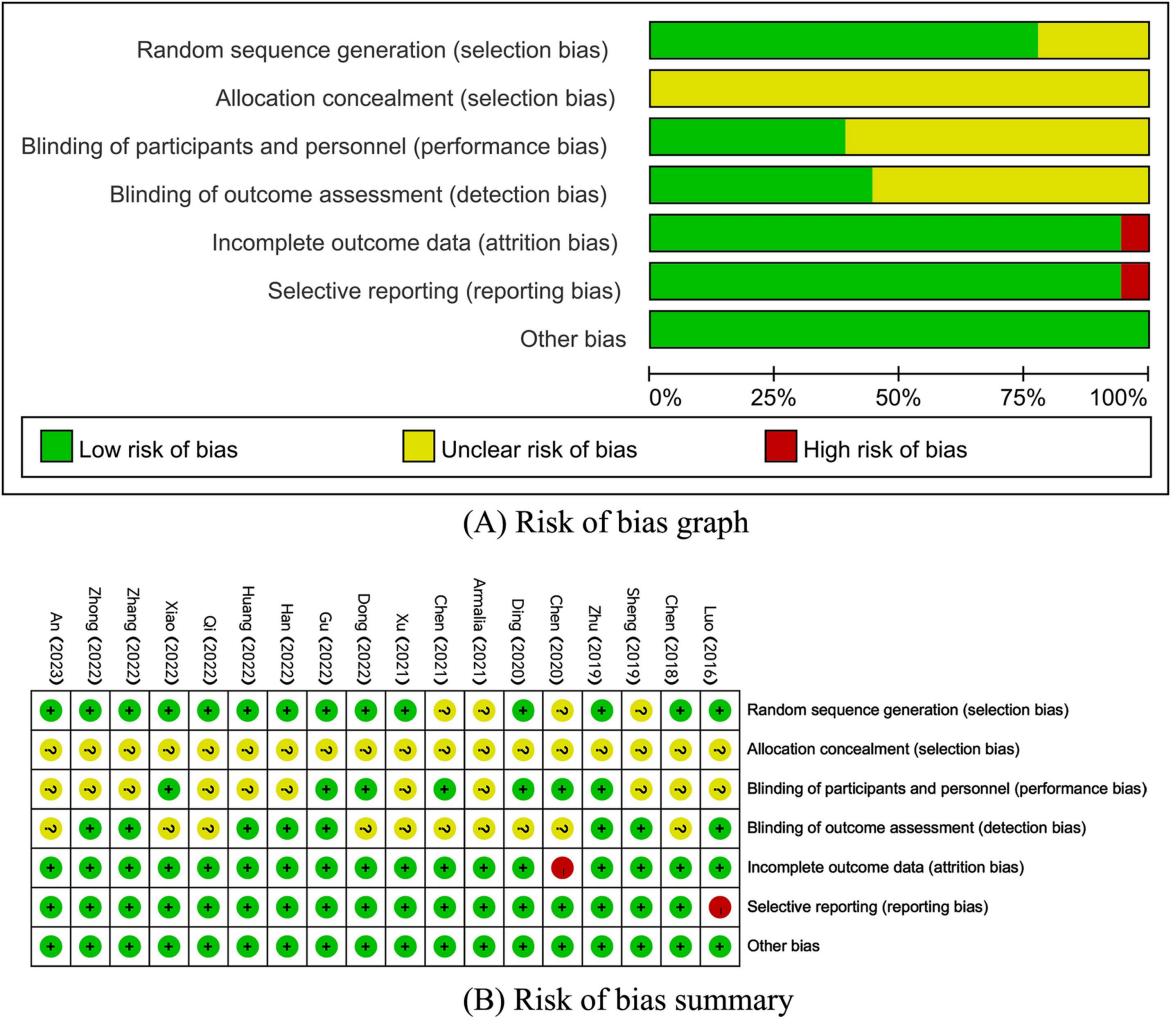
(A,B) Risk of bias assessment summary according to the Cochrane risk of bias tool. (A) Risk of bias graph; (B) Risk of bias summary.

Eighteen papers were included that mentioned randomization, but four of them did not explicitly mention the method of randomization. Seven papers blinded both subjects and experimenters, while eight papers blinded outcome assessors. One paper had a large and unbalanced number of missing persons at follow-up and did not take an appropriate approach to handling the missing values, and therefore was at high risk for ‘incomplete outcome data’. One paper did not report the prespecified outcome indicator, which resulted in a high risk of selective reporting (Figure 2).

### 3.3 Meta-analysis results

#### 3.3.1 Pittsburgh sleep quality index

**Figure 3:**
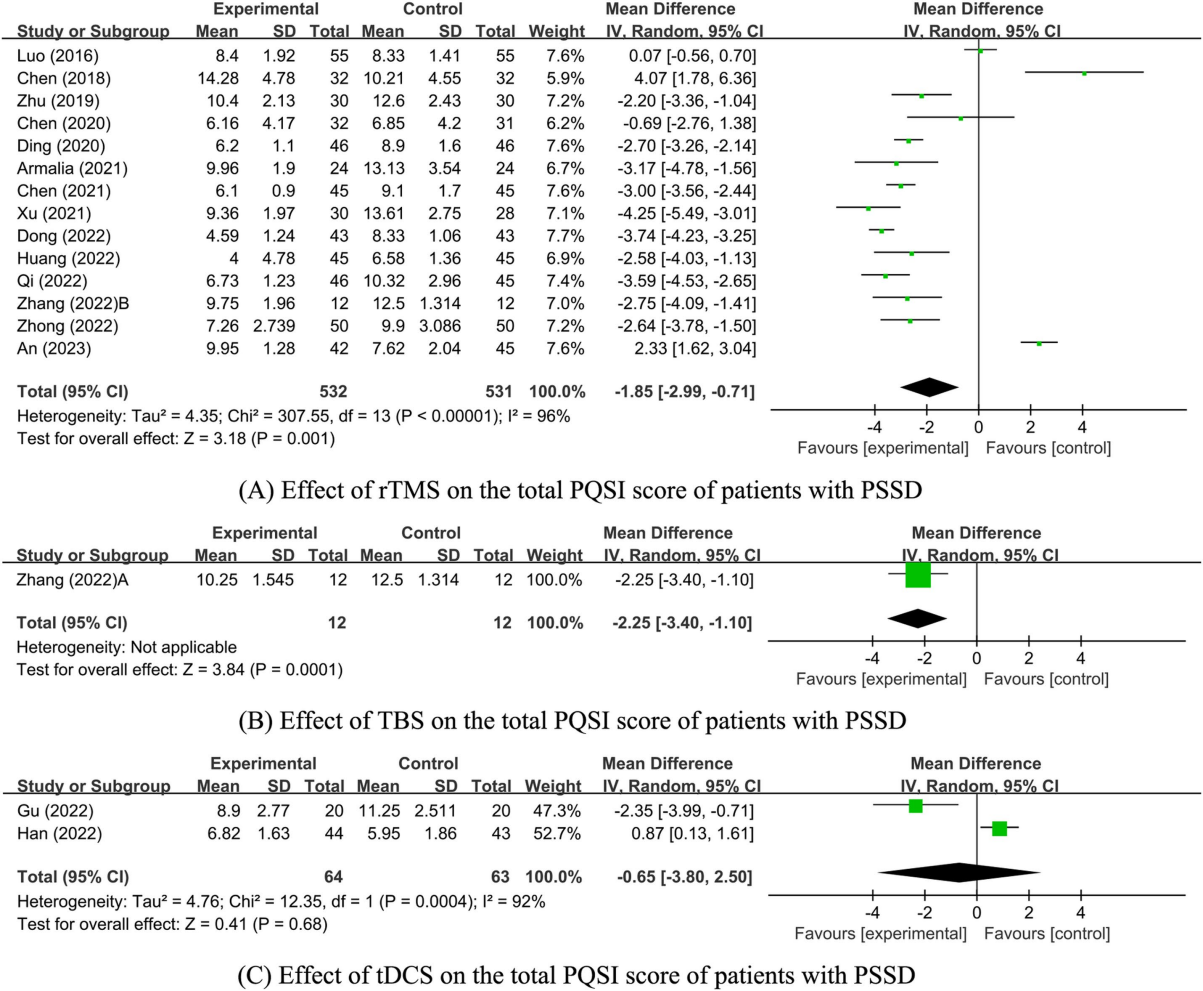
Effect of NIBS on the total PQSI score of patients with PSSD. (A) Effect of rTMS on the total PQSI score of patients with PSSD; (B) Effect of TBS on the total PQSI score of patients with PSSD; (C) Effect of tDCS on the total PQSI score of patients with PSSD.

Among the analyzed studies, 17 reported on the PSQI scores. However, one study (41) provided only the dimension-specific scores without the aggregate PSQI score, which prevented its inclusion in the overall analysis. Of the remaining 16 studies (32, 36, 38–40, 42–50, 52, 82) 14 involved rTMS stimulation (36, 39, 40, 42–50, 52, 82), one involved TBS stimulation (36), and two involved tDCS stimulation (32, 38). Due to differences in intervention methods, separate analyses were conducted. Since there was only one study on TBS, a descriptive analysis was performed, showing that PSQI scores in the TBS group were significantly lower than those in the control group (*p* = 0.0001). Meta-analysis results for the rTMS and tDCS groups revealed that PSQI scores in the rTMS group were significantly lower than those in the control group [MD = −1.85, 95% CI [−2.99, −0.71], *p* = 0.001]. In contrast, there was no significant difference between the tDCS group and the control group [MD = −0.65, 95% CI (−3.80, 2.50) *p* = 0.68; Figure 3].

Given the high heterogeneity between the rTMS and tDCS groups, further sensitivity and subgroup analyses were conducted to evaluate the stability of the meta-analysis results and identify the sources of heterogeneity. However, due to the small number of studies in the tDCS group (*n* ≤ 2), these analyses were not performed for this group. The heterogeneity in the tDCS group may stem from the varying precision of the tDCS interventions (one study used high-precision tDCS, while the other used standard tDCS). In the rTMS group, sensitivity analysis was conducted for each included study. The analysis revealed that excluding three highly heterogeneous studies An et al. (82), Luo et al. (39), and Dong (48) reduced heterogeneity (*I^2^* = 79%, *p* < 0.00001). The PSQI score of the rTMS experimental group remained significantly lower than that of the control group [MD = −2.44, 95% CI (−3.17, −1.72), *p* < 0.00001], indicating stable and reliable results.

The effect of rTMS on PSQI scores was further assessed through subgroup analysis by stimulation frequency. Employing a random-effects model (*p* < 0.00001, *I^2^* = 96%), this analysis found that both LF-rTMS and HF-rTMS groups had significantly lower PSQI scores than the control group [LF-rTMS: MD = -1.45, 95% CI (−2.77, −0.12), *p* = 0.03; HF-rTMS: MD = -2.95, 95% CI (−3.95, −1.95), *p* < 0.00001; Supplementary Figure S1A; Table 1]. Additional subgroup analyses differentiated the experimental and control groups into three categories based on rTMS interventions. These analyses revealed a significant reduction in PSQI scores for the rTMS combined with medication group compared to the medication-only group [MD = -2.86, 95% CI (−3.63, −2.10), *p* < 0.00001] and the sham or blank group [MD = -2.70, 95% CI (−3.17, −2.22), *p* < 0.00001]. However, the rTMS and medication-only groups did not show a significant difference in PSQI scores [MD = -0.66, 95% CI (−2.66, 1.34), *p* = 0.52; Supplementary Figure 1B; Table 2].

**Table 2:** Subgroup analysis of the effect of rTMS on PQSI scores in patients with PSSD.

Subgroup	Studies (N)	heterogeneity		MD [95%CI]	Z	*P*
		*I^2^*	*P*			
Different stimulation frequencies of rTMS:						
LF-rTMS	10 (36, 39, 40, 42, 44, 47, 49, 50, 52, 82)	0.96	*****	-1.45 [−2.77, −0.12]	2.14	0.03
HF-rTMS	3 (43, 46, 48)	0.81	0.006	-2.95 [−3.95, −1.95]	5.79	*****
Different rTMS interventions:						
A	6 (39, 40, 47, 49, 50, 82)	0.97	*****	-0.66 [−2.66, 1.34]	0.64	0.52
B	5 (42, 43, 45, 46, 48)	0.71	0.008	−2.86 [−3.63, −2.10]	7.31	*****
C	3 (36, 44, 52)	0	0.99	−2.70 [−3.17, −2.22]	11.21	*****

“*****” means: *p* < 0.00001; “−”: None. Subgroup A (E VS C): rTMS VS medication; Subgroup B (E VS C): rTMS+ medication VS medication; Subgroup C (E VS C): rTMS VS sham stimulation/blank group.

#### 3.3.2 Polysomnography

**Figure 4:**
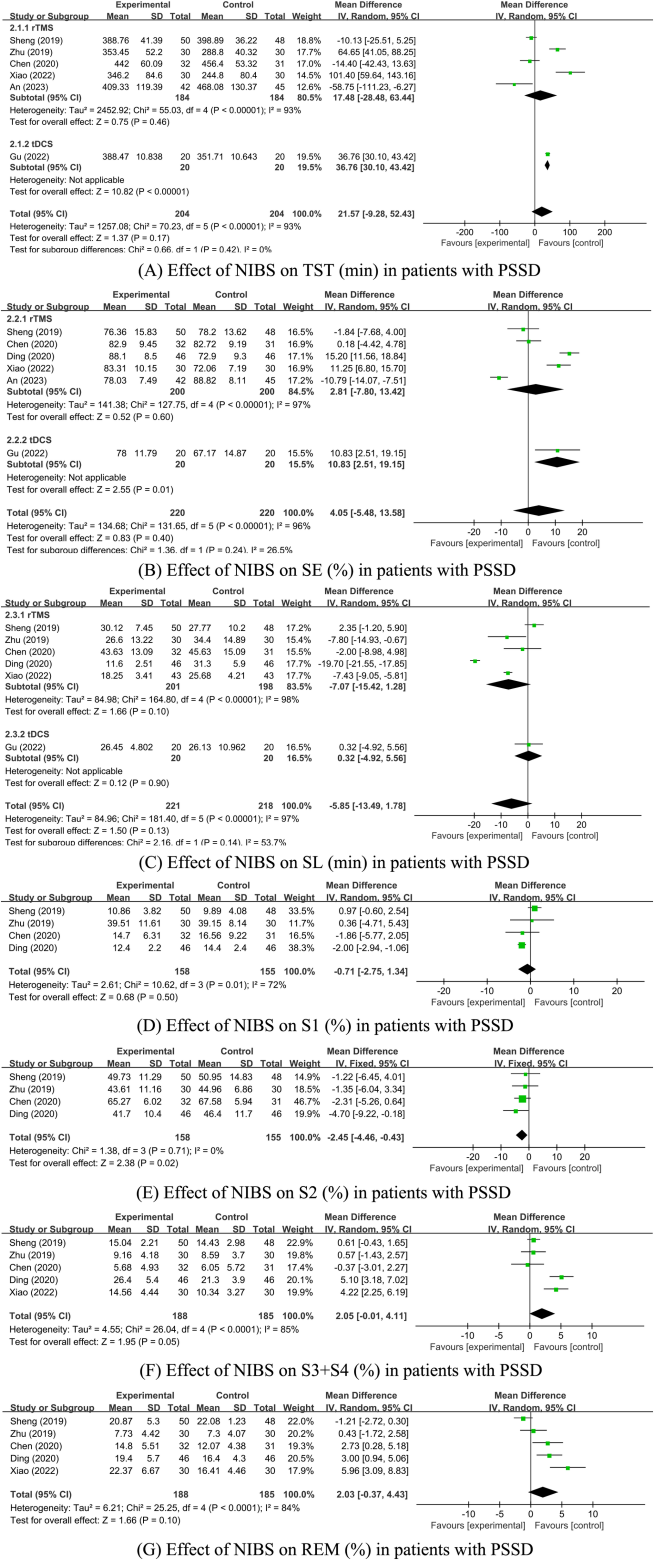
(A–G) Effect of NIBS on each sleep parameter of PSG in PSSD patients. Effect of NIBS on TST (min) in patients with PSSD; (B) Effect of NIBS on SE (%) in patients with PSSD; (C) Effect of NIBS on SL (min) in patients with PSSD; (D) Effect of NIBS on S1 (%) in patients with PSSD; (E) Effect of NIBS on S2 (%) in patients with PSSD; (F) Effect of NIBS on S3 + S4 (%) in patients with PSSD; (G) Effect of NIBS on REM (%) in patients with PSSD.

Seven studies were identified that reported sleep parameters using PSG. Among these, one study utilized tDCS and provided data on Total Sleep Time (TST), Sleep Efficiency (SE), and Sleep Latency (SL). The other studies employed rTMS. Due to the different intervention measures, the analyses were conducted separately. Since there was only one study on tDCS, a descriptive analysis indicated that the sleep parameters TST and SE in the tDCS group were significantly better than those in the control group (*p* < 0.05), while SL was not significantly improved (*p* > 0.05; Figure 4). The heterogeneity test results for the rTMS group showed that a fixed-effect model was adopted for S2% (*p* = 0.71, *I^2^* = 0%). A random-effects model was used for other sleep parameters. The meta-analysis results for the rTMS group showed no statistically significant differences in TST, SE, SL, S1%, and REM% between the two groups (*p* > 0.05). However, there were statistically significant differences in S2% and S3 + S4% between the groups (*p* < 0.05; Figure 4).

The inter-study heterogeneity of sleep parameters in the rTMS group remained substantial. Therefore, sensitivity and subgroup analyses were conducted to evaluate the stability of the meta-analysis results and identify the sources of heterogeneity. For studies with *I^2^* > 50% and more than two articles on sleep parameters, sensitivity analysis was conducted by sequentially excluding each study. The results showed that the heterogeneity of SE, SL, and S1% was significantly reduced, leading to the use of the fixed-effects model. The heterogeneity of the other three parameters did not significantly decrease, so the random-effects model was applied. For sleep parameters other than REM (%), excluding studies with high bias did not significantly alter the remaining meta-analysis results, indicating that the results were generally stable (see Table 3). However, after excluding the study by Sheng et al. (41) the REM (%) sleep parameter results changed, suggesting that these results are sensitive to the number of studies and lack robustness, thus requiring cautious interpretation. Sheng et al.’s study had a significant impact on the results, possibly because the proportion of REM sleep in the experimental group was significantly lower than in the control group, while other studies showed the experimental group had a higher REM proportion than the control group. This discrepancy may be related to the alternating high and low-frequency rTMS stimulation used in Sheng et al.’s study. Due to the continued high heterogeneity in some studies, further subgroup analysis was conducted to identify sources of heterogeneity.

**Table 3:** Sensitivity analysis of the effect of rTMS on sleep parameters of PSG in PSSD patients.

Parameters of sleep	Before excluding relevant studies			Exclusion studies	After excluding relevant studies				
	Effects model	MD [95%CI]	*P*		heterogeneity		Effects model	MD [95%CI]	*P*
					*I^2^*	*P*			
TST (min)	Random	17.48 [−28.48,63.44]	0.46	Sheng et al. (41) and Zhu et al. (42)	0.93	*****	Random	10.05 [−75.88,95.98]	0.82
SE (%)	Random	2.81 [−7.80,13.42]	0.60	Ding et al. (44), An et al. (82), and Xiao et al. (51)	0	0.59	Fixed	−0.59 [−4.21,3.02]	0.75
SL (min)	Random	−7.07 [−15.42,1.28]	0.10	Sheng et al. (41), Ding et al. (44), and Xiao et al. (51)	0.23	0.25	Fixed	−4.86 [−10.54,0.83]	0.09
S1 (%)	Random	−0.71 [−2.75,1.34]	0.50	Ding et al. (44)	0	0.42	Fixed	0.56 [−0.84,1.96]	0.43
S2 (%)	Fixed	−2.45 [−4.46,-0.43]	0.02	−	−	−	−	−	−
S3 + S4 (%)	Random	2.05 [−0.01,4.11]	0.05	Sheng et al. (41)	0.83	0.0005	Random	2.46 [−0.10,5.03]	0.006
REM (%)	Random	2.03 [−0.37,4.43]	0.10	Sheng et al. (41)	0.68	0.03	Random	2.89 [0.82,4.97]	0.005

TST, total sleep time; SE, Sleep efficiency; SL, sleep latency, refers to the time from lights out to sleep; S1 represents the first stage of sleep, S2 represents the second stage of sleep, S3 + S4 represents the deep sleep stage or slow-wave sleep, S1, S2, S3 + S4 are non-rapid eye movement (NREM) sleep stages, S1, S2, S3 + S4 are non-rapid eye movement (NREM) sleep stages. Rapid eye movement (REM) stands for rapid eye movement sleep. “*****” means: *p* < 0.00001.

Subgroup analyses were performed based on different TMS stimulation interventions (Table 4). The results indicated that the heterogeneity was reduced following the subgroup analysis. Subgroup analyses based on TMS stimulation intervention yielded varied outcomes: in subgroup A (rTMS vs. medication), no significant differences in TST, SE, SL, S1%, S2 (%), (S3 + S4)%, and REM% were found between the groups. In subgroup B (rTMS + medication vs. medication), all sleep parameters except for SL showed no significant variance. Conversely, in subgroup C (rTMS vs. sham/blank group), significant differences in SE, SL, S1%, S2 (%), (S3 + S4) %, and REM% were evident between the groups (Supplementary Figure S2; Table 4).

**Table 4:** Subgroup analysis of the effect of rTMS on PSG sleep parameters in PSSD patients.

Parameters of sleep	Subgroup	Studies(N)	Heterogeneity		MD [95%CI]	Z	*P*
			*I^2^*	*P*			
TST(min)	A	2 (41, 82)	0.67	0.08	−27.70 [−73.48, 18.08]	1.19	0.24
	B	3 (42, 43, 51)	0.93	*****	49.36 [−14.40, 113.13]	1.52	0.13
SE (%)	A	2 (41, 82)	0.85	0.009	−6.65 [−15.40, 2.09]	1.49	0.14
	B	2 (43, 51)	0.91	0.0007	5.73 [−5.12, 16.58]	1.04	0.3
	C	1 (44)	−	−	15.20 [11.56, 18.84]	8.18	*****
SL (min)	A	1 (41)	−	−	2.35 [−1.20, 5.90]	1.3	0.19
	B	3 (42, 43, 51)	0.10	0.33	−6.97 [−9.14, −4.80]	6.28	*****
	C	1 (39)	−	−	−19.70 [−21.55, −17.85]	20.84	*****
S1 (%)	A	1 (41)	−	−	0.97 [−0.60, 2.54]	1.21	0.22
	B	2 (42, 43)	0	0.82	0.92 [−0.58, 2.41]	1.2	0.23
	C	1 (44)	−	−	−2.00 [−2.94, −1.06]	4.17	****
S2 (%)	A	1 (41)	−	−	−1.22 [−6.45, 4.01]	0.46	0.65
	B	2 (42, 43)	0	0.73	−2.04 [−4.54, 0.46]	1.6	0.11
	C	1 (44)	−	−	−4.70 [−9.22, −0.18]	2.04	0.04
S3 + S4 (%)	A	1 (41)	−	−	0.61 [−0.43, 1.65]	1.15	0.25
	B	3 (42, 43, 51)	0.80	0.007	1.55 [−1.24, 4.35]	1.09	0.28
	C	1 (44)	−	−	5.10 [3.18, 7.02]	5.19	*****
REM (%)	A	1 (41)	−	−	−1.21 [−2.72, 0.30]	1.57	0.12
	B	3 (42, 43, 51)	0.78	0.01	2.92 [−0.14, 5.99]	1.87	0.06
	C	1 (44)	−	−	3.00 [0.94, 5.06]	2.85	0.04

Subgroup A, rTMS VS medication; Subgroup B, rTMS+ medication VS medication; Subgroup C, rTMS VS sham stimulation/blank group. “*****” means: *p* < 0.00001; “****” means: *p* < 0.0001; “−”: None.

#### 3.3.3 Secondary outcome measures

##### 3.3.3.1 HAMD-17 score

**Figure 5:**
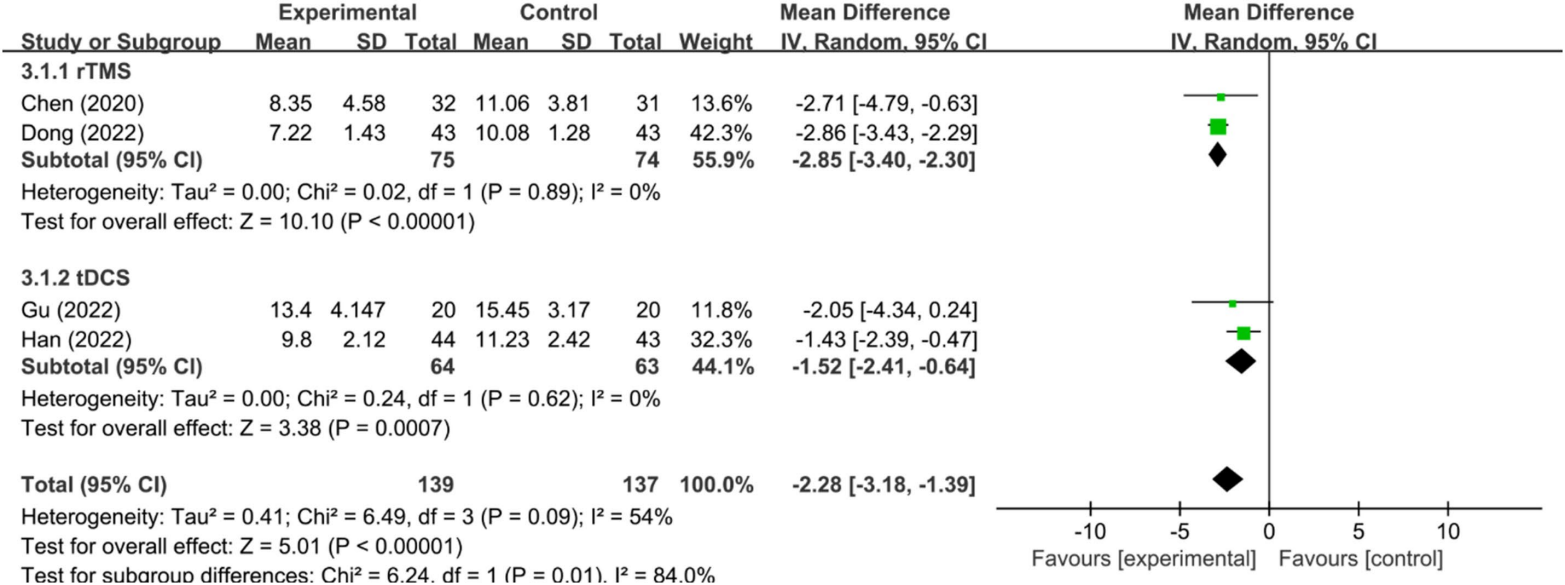
Effect of NIBS on HAMD-17 scores in patients with PSSD.

Five RCTs (32, 36, 38, 43, 48) assessed HAMD-17 scores. In one of these studies (36), the HAMD-17 scores were non-normally distributed, rendering the mean and standard deviation inextractable and precluding meta-analysis integration. Among the remaining four studies (32, 38, 43, 48), significant heterogeneity was detected (*I^2^* = 54%, *p* < 0.1), necessitating the use of a random-effects model. The meta-analysis revealed that the HAMD-17 scores in the NIBS group were significantly lower than those in the control group [MD = −2.28, 95% CI (−3.18, −1.39), *p* < 0.00001]. Sensitivity analysis, excluding the study by Dong et al. (48), resulted in negligible heterogeneity (*I^2^* = 0%, *p* = 0.62) and maintained the statistical significance of the intervention effect under a fixed-effects model [MD = −1.70, 95% CI (−2.51, −0.89), *p* < 0.0001], affirming the reliability of the results. Further subgroup analyses across different NIBS modalities, including rTMS and tDCS, indicated that the HAMD-17 scores were significantly lower in both rTMS and tDCS groups compared to the control group [MD = −2.85, 95% CI (−3.40, −2.30), *p* < 0.00001; MD = −1.52, 95% CI (−3.41,-0.64), *p* = 0.0007; Figure 5].

##### 3.3.3.2 Brain-derived neurotrophic factor

**Figure 6:**
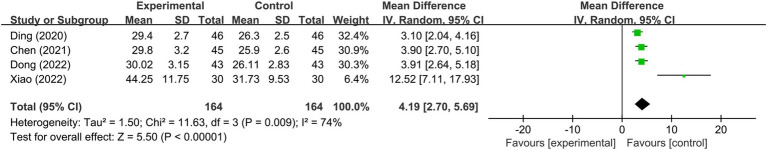
Effect of rTMS on serum BDNF levels in patients with PSSD.

In the context of serum BDNF levels, four RCTs (44, 46, 48, 51) were analyzed, all employing rTMS as the stimulation modality. A meta-analysis utilizing a random-effects model indicated substantial heterogeneity (*I^2^* = 74%, *p* < 0.1) but revealed that serum BDNF levels in the NIBS group were significantly higher compared to the control group [MD = 4.19, 95%CI = (2.70, 5.69), *p* < 0.00001; Figure 6]. Sensitivity analysis, after excluding the study by Xiao et al. (51), showed a negligible inter-study heterogeneity (*I^2^* = 0%, *p* = 0.52), and the effect remained statistically significant under a fixed-effects model [MD = 3.58, 95% CI (2.91, 4.26), *p* < 0.00001], suggesting the robustness of these findings. One possible reason for this difference is that Xiao (51) experienced a stimulation intensity of 120%MT, whereas the other four studies used 80%MT.

##### 3.3.3.3 Adverse reactions

**Figure 7:**
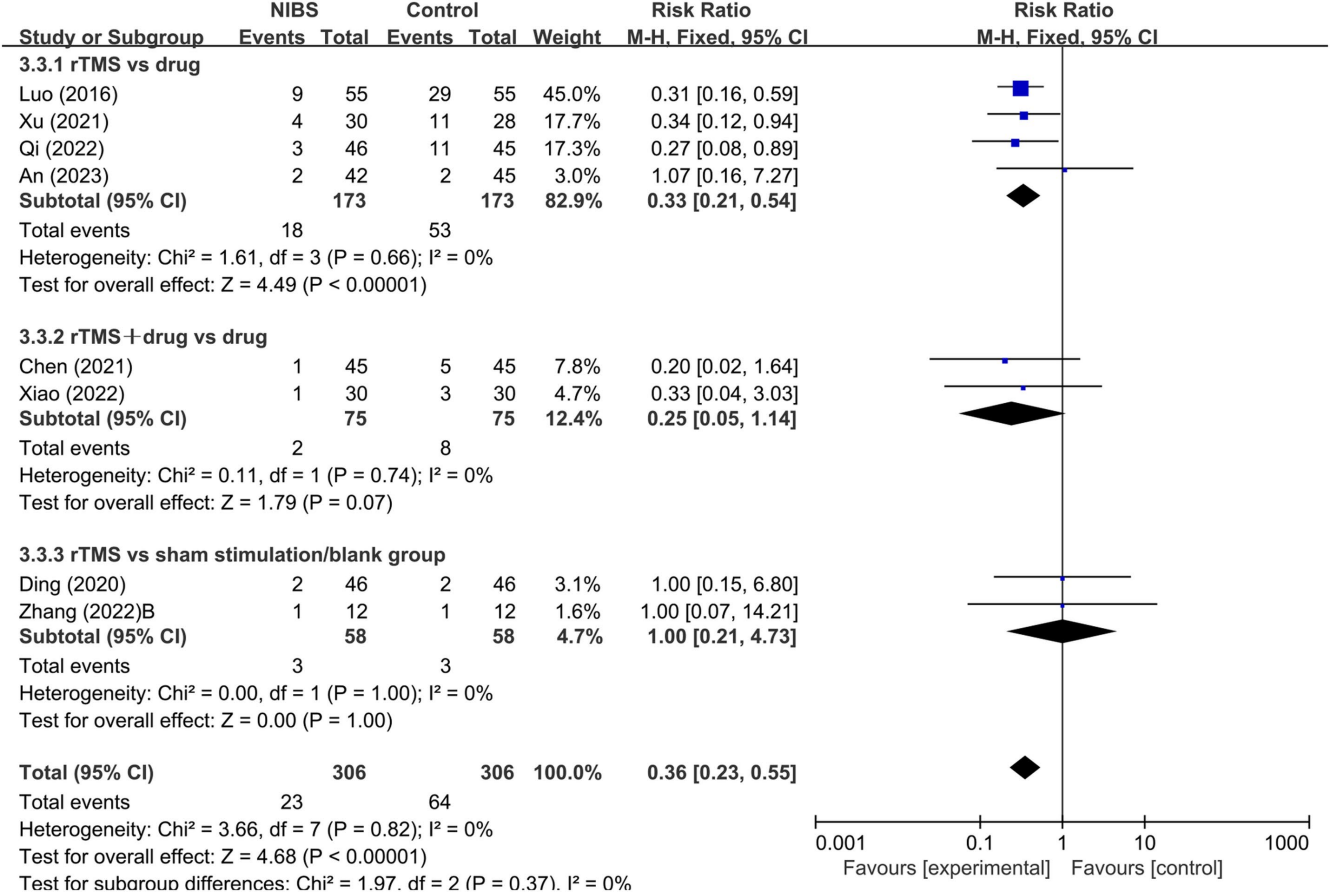
Forest plot of adverse effects in patients with PSSD treated with rTMS.

Eight RCTs (36, 39, 44, 46, 47, 50, 51, 82) reported on adverse reactions, utilizing rTMS as the mode of stimulation. A fixed-effects model meta-analysis (*I^2^* = 0%, *p* > 0.1) demonstrated that the incidence of adverse reactions in the NIBS group was significantly lower than in the control group [RR = 0.36, 95% CI (0.23, 0.55), *p* < 0.0001]. Subgroup analysis, based on different rTMS interventions, indicated a significantly lower incidence of adverse reactions in the rTMS group compared to the medication group (*p* < 0.0001). However, no significant difference was found in the incidence of adverse reactions between the rTMS combined with the medication group and the medication-only group (*p* > 0.05), as well as between the rTMS group and the sham stimulation/blank group (*p* > 0.05; Figure 7).

### 3.4 Publication bias

**Figure 8:**
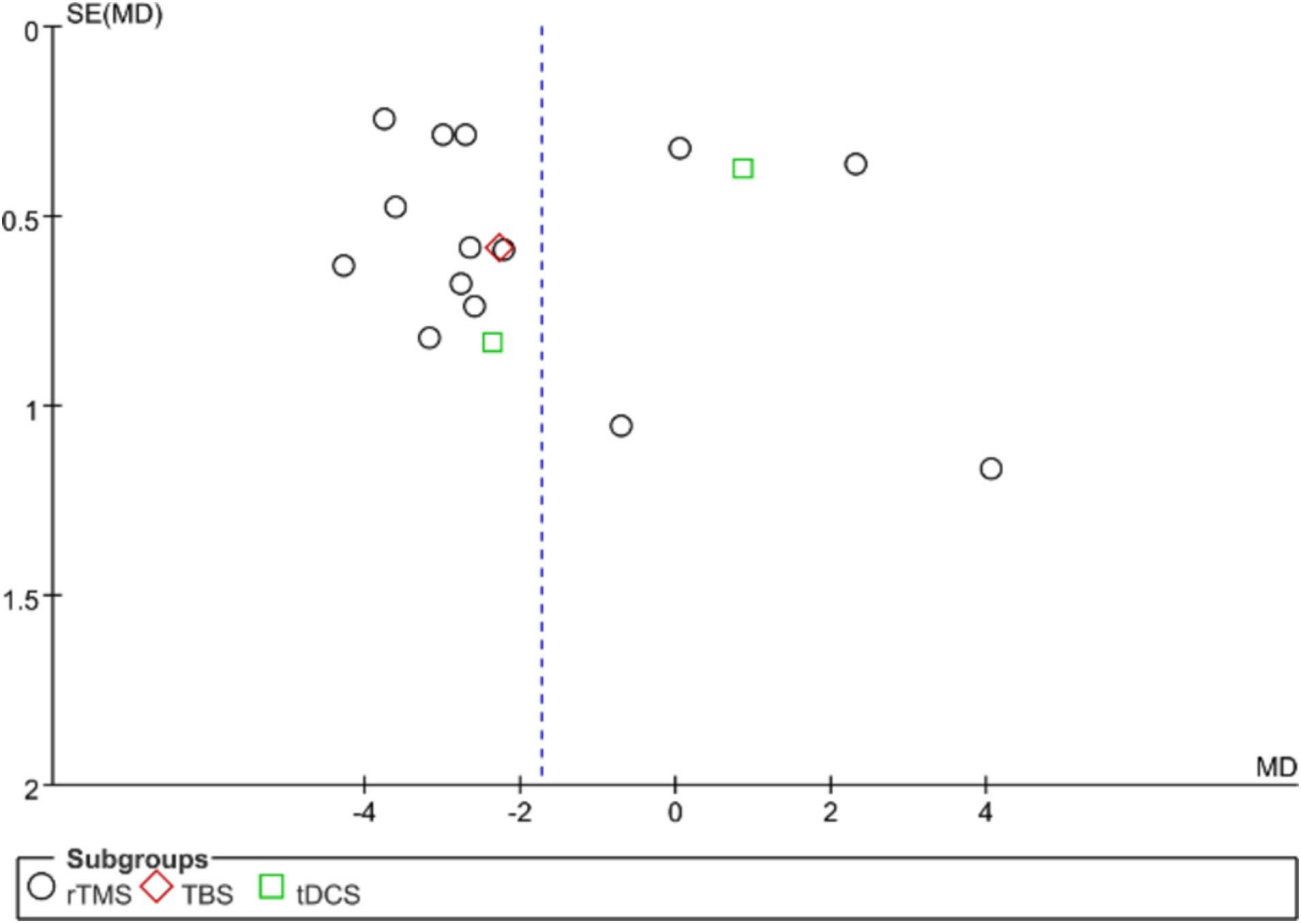
Shows an inverted funnel plot using PSQI scores as an outcome indicator.

Publication bias in the analyzed studies was assessed using the PQSI scores of the primary outcome measures, with the associated funnel plot presented in Figure 8. Although the inverted funnel plot exhibited no apparent asymmetry, it did not completely rule out the possibility of publication bias. Egger’s test (*p* = 0.701) indicated an absence of significant publication bias across the studies. Publication bias assessment for other outcome measures was not conducted due to the inclusion of fewer than nine studies.

## 4 Discussion

NIBS techniques have been employed in the treatment of post-stroke sleep disorders (PSSD). However, a comprehensive meta-analysis of recent randomized controlled trials (RCTs) evaluating their efficacy remains absent in the literature. The primary objective of this study was to assess the therapeutic effect of NIBS on PSSD. Our meta-analysis consolidates the current evidence, providing a robust validation of the significant therapeutic benefits of NIBS in improving sleep quality in post-stroke patients. This synthesis of findings offers valuable insights into the optimal application of NIBS in clinical practice for the management of PSSD.

### 4.1 Effect of NIBS technique on PQSI total score of PSSD patients

The results of this study showed that the PSQI scores in the rTMS and TBS experimental groups within NIBS were significantly lower than those in the control group (*p* < 0.05), consistent with the findings of Guo et al. (53). However, there was no statistically significant difference between the tDCS experimental group and the control group. Due to the limited number of existing studies, particularly concerning TBS and tDCS, the stability of the results may be affected. Furthermore, the heterogeneity observed in the meta-analysis indicates that the findings may be unstable and should be interpreted with caution. Future research is needed to provide further evidence-based support. Subgroup analysis revealed that both low-frequency rTMS (LF-rTMS) and high-frequency rTMS (HF-rTMS) significantly lowered total PSQI scores compared to the control group (*p* < 0.05), corroborating Guo et al.’s (53) results. Various treatment options were examined, and subgroup analysis of different intervention schemes revealed that rTMS improved sleep quality in PSSD patients, and combining rTMS with pharmacotherapy further enhanced the therapeutic effect. However, there was no significant difference in improving sleep quality between TMS stimulation and pharmacotherapy alone. The considerable heterogeneity among the subgroups might affect the reliability of the conclusions, so they should be interpreted with caution. A review of the original literature suggests that this heterogeneity may result from multiple factors, such as stimulation site, intensity, intervention duration, and combination with medication. However, due to the small number of included studies, there was insufficient sample size and statistical power for a multifactorial meta-regression analysis to further explore the heterogeneity among studies.

Sleep plays a crucial role in synaptic plasticity, a process fundamental to recovery following stroke or other cerebrovascular diseases (54). It is well-established that slow-wave sleep (SWS) supports neuroplasticity, facilitating the strengthening and pruning of synapses essential for post-stroke recovery (55). NIBS, especially rTMS and TBS, has emerged as a promising approach in modulating these *in-vivo* circuits, potentially enhancing plasticity through the targeted activation of cortical networks during sleep. This neuro-modulatory role of NIBS may explain the observed improvements in PSQI scores, as these techniques could be influencing the underlying sleep mechanisms critical for brain plasticity.

TMS and TES, has emerged as a promising approach for modulating neuroplasticity by targeting cortical circuits involved in sleep regulation and post-stroke recovery. Evidence indicates that rTMS can extend synaptic plasticity, regulate the connectivity strength between brain regions, modulate cortical excitability, and influence neurotransmitter dynamics (18). Synaptic plasticity, broadly defined, refers to the capacity for adjusting the strength of connections between synapses and encompasses characteristics such as LTP, output transferability, associativity, cooperativity, persistence, LTD, and short-term synaptic plasticity. rTMS primarily affects short-term synaptic plasticity, enhancing the certainty of synaptic transmission while balancing excitatory and inhibitory activity within the cortex. This modulation reduces the excitatory effects associated with depression and enhances inhibitory mechanisms, facilitating the spatiotemporal characteristics of neural activity and generating synchronized oscillations within the cortical-thalamic network, ultimately leading to improved sleep outcomes (56). In clinical practice, the standard application protocol involves LF-rTMS targeting the right dorsolateral prefrontal cortex (DLPFC) to induce hyperpolarization of cortical neurons, thereby decreasing the excitability of this region and normalizing aberrant connections between the prefrontal cortex and other distant sites (57). Moreover, extensive research has validated the role of the hippocampus in cognition and learning. Recent findings suggest that rTMS may promote neurogenesis in the hippocampus and alleviate neuronal damage, thereby improving chronic insomnia (58).

Currently, research on TMS for PSSD primarily focuses on rTMS, with fewer studies examining TBS. However, TBS is recognized for its brief yet enduring impact, with efficacy that matches or surpasses rTMS in various domains. Thus, the role of TBS in PSSD warrants further exploration. The underlying mechanisms of TBS are fundamentally consistent with those of traditional rTMS, involving changes at both the genetic and protein levels. TBS is characterized by a high treatment frequency, low stimulation intensity, and short duration.The main distinction between TBS and traditional rTMS lies in TBS’s ability to induce changes in cortical excitability through short-duration stimulation (40 to 190 s), with effects lasting for at least 20 to 30 min post-stimulation. There are two common TBS paradigms:Intermittent TBS (iTBS) can induce long-term potentiation (LTP) effects, thereby enhancing cortical xcitability.Continuous TBS (cTBS) induces long-term depression (LTD), which suppresses cortical excitability. TBS acts more rapidly than traditional rTMS treatment, requiring shorter stimulation durations and lower intensities to achieve changes in cortical excitability comparable to those of rTMS, with effects lasting for a similar duration (59). Therefore, the role of TBS in PSSD warrants further exploration.

Studies have shown that tDCS enhances sleep quality in individuals with insomnia, promotes an increase in non-rapid eye movement (NREM) slow-wave sleep, and helps regulate the sleep–wake cycle (60). However, the generalizability of tDCS in improving sleep is debated, with some researchers positing its effectiveness in younger individuals, potentially due to age-related sleep mechanism variations. With only two tDCS studies included, exhibiting considerable heterogeneity, the findings remain tentative, underlining the need for more research to solidify the evidence. tDCS can significantly affect neuronal activity by altering membrane polarity and cortical excitability. tDCS enhances synaptic plasticity and modulates the excitatory/inhibitory balance of the cortex. GABA, the primary inhibitory neurotransmitter, works in concert with the excitatory neurotransmitter glutamate to influence neuronal activity and functional plasticity. Studies have shown that insomnia patients exhibit significantly lower GABA levels compared to healthy individuals, particularly in the occipital cortex and anterior cingulate cortex (61). Furthermore, tDCS stimulation can regulate GABA and glutamate concentrations: anodal tDCS increases GABA levels, while cathodal tDCS suppresses neural transmission through glutamate modulation (62). Additionally, tDCS improves local cerebral blood flow (CBF) regulation, ensuring stable blood supply and oxygenation to neurons. Research suggests that tDCS promotes increased cerebral blood flow through the induction of vasodilatory factors such as nitric oxide (NO) and vascular endothelial growth factor (VEGF) (63). In insomnia patients, tDCS enhances cerebral oxygenated hemoglobin and other hemodynamic markers, thereby improving local CBF (64).

Despite our findings indicating that rTMS significantly improves sleep quality, there are several limitations. The heterogeneity among studies, possibly due to differences in experimental design, stimulation parameters, and patient characteristics, might affect the reproducibility and consistency of rTMS’s therapeutic effects. Furthermore, the limited number of included studies precluded a multifactorial meta-regression analysis to explore potential confounding factors, restricting the generalizability of our conclusions. While both LF-rTMS and HF-rTMS show promise, their optimal parameters remain unclear, necessitating further research. Additionally, our study focused primarily on short-term effects, leaving the long-term efficacy and safety of rTMS unknown. Future research should include long-term follow-ups to assess sustained efficacy and potential side effects. In summary, although this study provides preliminary evidence of rTMS improving sleep quality, the results should be interpreted with caution, and further research is needed to address existing limitations.

### 4.2 Effect of NIBS technique on each sleep parameter of PSG in PSSD patients

Research indicates that patients with PSSD have altered sleep architecture and diminished sleep quality, notably with increased N1 and N2 sleep stages (S1% and S2%) and reduced Total Sleep Time (TST), Sleep Efficiency (SE), and slow-wave sleep stages (N3, represented as S3 + S4%), as well as shorter Rapid Eye Movement (REM) sleep duration (65). The present study showed that tDCS stimulation could improve TST and SE but not sleep latency (SL) in PSSD patients. Similarly, rTMS notably decreased S2 and S3 + S4 sleep stages percentages in this patient group. Yet, no statistically significant variations were observed between groups concerning TST, SE, SL, S1%, and REM%, aligning with the findings of Gao et al. (66).

Research has shown that the hyperarousal state in patients with sleep disorders leads to the overactivation of both the hypothalamic–pituitary–adrenal (HPA) axis and the hypothalamic–pituitary-thyroid (HPT) axis. Serum cortisol and adrenocorticotropic hormone (ACTH) serve as critical indicators for HPA axis activity, while hypersensitive thyroid-stimulating hormone (hTSH), free triiodothyronine (FT3), and free thyroxine (FT4) levels correlate with the degree of HPT axis activation (67, 68). Studies have demonstrated that rTMS effectively reduces serum levels of cortisol, hTSH, FT3, FT4, and ACTH, thereby mitigating the hyperarousal state and improving sleep quality in patients with insomnia (24). Furthermore, tDCS modulates cortical and subcortical electrical activity, promoting the generation of slow-wave sleep (SWS), which is essential for sleep maintenance and memory consolidation. Insomnia patients exhibit a reduction in SWS and an increase in light sleep frequency in polysomnography (PSG) assessments. By inducing deep sleep frequencies (0.75 Hz) in brain waves, tDCS helps stabilize sleep, increase spindle count, and enhance electroencephalographic power (69), ultimately improving sleep quality and total sleep duration.

Subgroup analysis of rTMS disclosed significant differences in sleep parameters (SE, SL, S1%, S2%, S3 + S4%, REM%) between the rTMS group and the sham/blank group. However, there were no statistically significant differences in any sleep parameters between the rTMS group and the medication group. Similarly, apart from SL, no significant differences were observed between the rTMS combined with medication group and the medication group. Therefore, rTMS did not significantly improve sleep parameters compared to medication alone, nor did combining rTMS with medication offer substantial additional benefits. Despite reduced heterogeneity from subgroup analyses, some inconsistencies remained, possibly influenced by the rTMS treatment specifics like combined medication, frequency, site, intensity, and duration. The limited number of studies for each PSG-derived sleep parameter and the small sample size contributed to the instability of the results, highlighting the need for further research to substantiate these findings.

In this study, we found that rTMS and tDCS stimulation had varying effects on sleep quality and architecture in patients with PSSD. However, these results might be influenced by multiple interacting factors, especially the stimulation target area and concurrent medication treatment. Firstly, the choice of target area plays a crucial role in NIBS treatment. Different target areas can result in different therapeutic outcomes. For instance, studies have shown that rTMS targeting the dorsolateral prefrontal cortex (DLPFC) on either side is more effective for treating chronic insomnia than other areas, significantly improving sleep quality, sleep beliefs, and attitudes (70, 71). This may be because the DLPFC is central to the neural mechanisms of cognitive and emotional control, helping to reduce the impact of negative emotions and enhance the recognition of positive emotions (72). The variation in target areas in the studies we analyzed may be a source of result heterogeneity.

Notably, the role of pharmacotherapy in modulating motor cortex activity and plasticity is crucial. Acute drug intake, particularly those affecting GABAergic and glutamatergic transmission, can enhance or inhibit the effects of NIBS techniques, leading to variable outcomes in sleep quality and neuroplasticity. Conversely, chronic exposure to certain medications may alter brain organization and plasticity, complicating the interpretation of NIBS efficacy in clinical settings. Our study found that while rTMS combined with medication did not significantly improve sleep parameters compared to medication alone, this could be because the medication itself is highly effective, potentially masking the additional benefits of rTMS. Some studies have shown that estazolam combined with LF-rTMS is particularly effective for chronic insomnia, especially in improving sleep quality and memory function in elderly patients. Estazolam helps patients fall asleep quickly but may cause residual sedation the next day, affecting memory, attention, and cognitive function (73). In contrast, LF-rTMS can protect neurons and synaptic function, improving memory and cognitive function, thereby enhancing daily living abilities, reducing the economic and psychological burden on families, and avoiding the waste of medical resources. Therefore, different medication regimens and patient responses to medication might also contribute to the heterogeneity of study results (73).

### 4.3 Effects of NIBS technique on secondary outcomes in PSSD patients

This study highlights the effectiveness of NIBS in ameliorating the depressive symptoms of patients with PSSD. Subgroup analyses reveal that both rTMS and tDCS are effective in improving mood in PSSD patients, aligning with the 2023 network meta-analysis findings (74). In the context of stroke, a significant correlation exists between mood and sleep disorders, with patients exhibiting depression and anxiety more prone to insomnia, which can exacerbate their mental health conditions (50). NIBS impacts mood by modulating the excitability and inhibition of cortical neurons, influencing brain region activities, neurotransmitter release, and ultimately enhancing mood in patients with post-stroke sleep disorders (31, 75).

The findings of this study indicate that rTMS may enhance sleep quality by elevating serum BDNF levels. BDNF, crucial for sleep regulation, can be adversely affected by prolonged sleep deprivation, leading to reduced levels (76). Conversely, elevated BDNF levels correlate with improvements in NREM sleep, increased slow wave activity, and extended N3 and REM sleep phases (77). Furthermore, rTMS has been demonstrated to raise serum BDNF levels in patients suffering from depression and sleep disorders (23, 78). This stimulation improves synaptic plasticity, augments the release of neurotransmitters such as dopamine and glutamate, and activates pathways including cAMP response element binding protein (CREB) and tyrosine receptor kinase B (TrkB), thus fostering BDNF synthesis and release (79). Nonetheless, the impact of rTMS on serum BDNF levels remains debated, with discrepancies potentially arising from variations in the timing of BDNF measurements and the rTMS frequency used (80). Additionally, while tDCS has been shown to increase serum BDNF levels in stroke patients (81), its efficacy in treating patients with PSSD remains underexplored. Therefore, whether tDCS can improve sleep quality in these patients by increasing BDNF levels warrants further investigation.

The study also indicates a more favorable safety profile for rTMS compared to pharmacotherapy, with fewer adverse reactions reported. However, the literature on tDCS in PSSD treatment is limited, and its safety profile in PSSD patients requires more comprehensive investigation.

This study has several limitations: Firstly, the limited number of studies on TBS and tDCS, coupled with heterogeneity among the studies, necessitates further validation and replication of results. Secondly, the duration of interventions varied across the studies. Thirdly, variations in the frequency of NIBS and the locations of stimulation sites could have biased the results. Additionally, the impact of NIBS on different stimulation sites and across age groups was not comprehensively analyzed. Fourthly, the predominance of literature in Chinese with insufficient corresponding English literature may have led to selection bias. Finally, the long-term effects of NIBS on PSSD were not adequately examined.

In conclusion, NIBS appears to be a promising treatment for PSSD, showing improvements in sleep quality and structure, depression symptoms, and levels of brain-derived neurotrophic factor in PSSD patients. However, the reliability of these findings is compromised by the small sample size, variation in intervention protocols, and differences in stimulation frequency, site, duration, and participant ages. To substantiate these preliminary findings, future research should focus on expanding the sample size and conducting high-quality multicenter randomized controlled trials. This approach will aid in establishing effective clinical treatments for PSSD and in identifying the optimal treatment modalities. Additionally, the therapeutic potential of TBS and tDCS for PSSD warrants further investigation.

## Data Availability

The original contributions presented in the study are included in the article/Supplementary material, further inquiries can be directed to the corresponding author.
