# Supplementary material for: Efficacy of non-invasive brain stimulation for post-stroke sleep disorders: a systematic review and meta-analysis
Source: Front Neurol. 2024 Oct 30;15:1420363. doi: 10.3389/fneur.2024.1420363 (PMC11557329; doi:10.3389/fneur.2024.1420363)
Supplement: Supplementary file 1 [file Data_Sheet_1.docx]

Supplementary Material 1

# search strategy

Final Search Record:2023-11-22

1.Database: **CNKI**

Date: 2023-11-22

Number of results:88

Search strategy:

( SU=(脑卒中 + 中风 + 脑血管疾病 + 脑血管意外 + 脑出血 + 脑梗死 + 脑血栓 + 偏瘫) AND SU=(睡眠障碍 + 睡眠 + 失眠) AND SU=(非侵入性脑刺激 + 无创脑刺激 + 经颅电刺激 + 经颅直流电刺激 + 经颅交流电刺激 + 经颅随机噪声刺激 + 经颅磁刺激 + 重复经颅磁刺激 +θ 爆发刺激 + TES + tDCS + tACS + tRNS + TMS + rTMS + TBS))

2.Database: **wanfang**

Date: 2023-11-22

Number of results:131

Search strategy:

( SU=(脑卒中 + 中风 + 脑血管疾病 + 脑血管意外 + 脑出血 + 脑梗死 + 脑血栓 + 偏瘫) AND SU=(睡眠障碍 + 睡眠 + 失眠) AND SU=(非侵入性脑刺激 + 无创脑刺激 + 经颅电刺激 + 经颅直流电刺激 + 经颅交流电刺激 + 经颅随机噪声刺激 + 经颅磁刺激 + 重复经颅磁刺激 +θ 爆发刺激 + TES + tDCS + tACS + tRNS + TMS + rTMS + TBS))

3.Database: **VIP**

Date: 2023-11-22

Number of results:82

Search strategy:

( SU=(脑卒中 + 中风 + 脑血管疾病 + 脑血管意外 + 脑出血 + 脑梗死 + 脑血栓 + 偏瘫) AND SU=(睡眠障碍 + 睡眠 + 失眠) AND SU=(非侵入性脑刺激 + 无创脑刺激 + 经颅电刺激 + 经颅直流电刺激 + 经颅交流电刺激 + 经颅随机噪声刺激 + 经颅磁刺激 + 重复经颅磁刺激 +θ 爆发刺激 + TES + tDCS + tACS + tRNS + TMS + rTMS + TBS))

3.Database: **CBM(SinoMed)** **(China Biology Medicine disc, CBMdisc)**

Date: 2023-11-22

Number of results:92

Search strategy:

1 "卒中"[不加权:扩展] 253030

2 "脑卒中"[常用字段:智能] OR "卒中"[常用字段:智能] OR "中风"[常用字段:智能] OR "脑血管疾病"[常用字段:智能] OR "脑血管意外"[常用字段:智能] OR "脑出血"[常用字段:智能] OR "脑梗死"[常用字段:智能] OR "脑血栓"[常用字段:智能] OR "偏瘫"[常用字段:智能] 550272

3 (#2) OR (#1) 550272

4 "经颅磁刺激"[不加权:扩展] OR "经颅直流电刺激"[不加权:扩展] 4763

5 "非侵入性脑刺激”[常用字段:智能] OR “无创脑刺激”[常用字段:智能] OR “经颅电刺激”[常用字段:智能] OR “经颅直流电刺激”[常用字段:智能] OR “经颅交流电刺激”[常用字段:智能] OR “经颅随机噪声刺激”[常用字段:智能] OR “经颅磁刺激”[常用字段:智能] OR “重复经颅磁刺激”[常用字段:智能] OR “θ爆发刺激”[常用字段:智能] OR “TES”[常用字段:智能] OR “tDCS”[常用字段:智能] OR “tACS”[常用字段:智能] OR “tRNS”[常用字段:智能] OR “TMS”[常用字段:智能] OR “rTMS”[常用字段:智能] OR “TBS”[常用字段:智能] 8215

6 (#5) OR (#4) 8215

7 "入睡和睡眠障碍"[不加权:扩展] OR "睡眠"[不加权:扩展] 52408

8 "睡眠障碍"[常用字段:智能] OR "睡眠"[常用字段:智能] 0R "失眠"[常用字段:智能]57897

9 (#8) OR (#7) 79343

10 (#9) AND (#6) AND (#3) 92

5.Database: **PubMed (MEDLINE)**

Date: 2023-11-22

Number of results: 37

Search strategy:

((("Stroke"[Mesh]) OR ("stroke*"[Title/Abstract] OR "apoplexy"[Title/Abstract] OR "cerebrovascular"[Title/Abstract] OR "hemiplegia"[Title/Abstract] OR "cerebral hemorrhage"[Title/Abstract] OR "cerebral infarction"[Title/Abstract] OR "cerebral stroke*"[Title/Abstract] OR "cerebral vascular accident*"[Title/Abstract] OR "brain vascular accident*"[Title/Abstract] OR "CVA"[Title/Abstract])) AND (("Transcranial magnetic stimulation"[Mesh] OR "transcranial direct current stimulation"[Mesh]) OR ("non-invasive brain stimulation"[Title/Abstract] OR "noninvasive brain stimulation"[Title/Abstract] OR "transcranial electrical stimulation"[Title/Abstract] OR "transcranial direct current stimulation"[Title/Abstract] OR "transcranial alternating current stimulation"[Title/Abstract] OR "transcranial random noise stimulation"[Title/Abstract] OR "transcranial magnetic stimulation"[Title/Abstract] OR "repetitive transcranial magnetic stimulation"[Title/Abstract] OR "theta burst stimulation"[Title/Abstract] OR "TES"[Title/Abstract] OR "tDCS"[Title/Abstract] OR "tACS"[Title/Abstract] OR "tRNS"[Title/Abstract] OR "TMS"[Title/Abstract] OR "rTMS"[Title/Abstract] OR "TBS"[Title/Abstract]))) AND (("sleep"[Mesh] OR "Sleep Wake Disorders"[Mesh]) OR ("Sleep*"[Title/Abstract] OR "Sleep Disorder*"[Title/Abstract] OR "Sleep Wake Disorder"[Title/Abstract] OR "Sleep-Related Neurogenic Tachypnea*"[Title/Abstract] OR "Sleeper Syndrome*"[Title/Abstract] OR "Dyssomnia*"[Title/Abstract] OR "Nocturnal Eating Drinking Syndrome*"[Title/Abstract] OR "Sleep Initiation and Maintenance Disorders"[Title/Abstract] OR "Insomnia"[Title/Abstract] OR "Disorders of Initiating and Maintaining Sleep"[Title/Abstract] OR "DIMS"[Title/Abstract] OR "Sleeplessness"[Title/Abstract] OR "Early Awakening"[Title/Abstract] OR "parasomnia*"[Title/Abstract] OR "hypersomnia"[Title/Abstract] OR "Circadian"[Title/Abstract] OR "restless leg syndrome"[Title/Abstract] OR "periodic limb"[Title/Abstract] OR "polysomnography"[Title/Abstract] OR "narcolepsy"[Title/Abstract] OR "somnolence"[Title/Abstract]))

6.Database: **web of science**

Date: 2023-11-22

Number of results:41

Search strategy:

#1 TS=(stroke* OR apoplexy OR cerebrovascular OR hemiplegia OR cerebral hemorrhage OR cerebral infarction OR cerebral stroke* OR cerebral vascular accident* OR brain vascular accident* OR CVA) 320009

#2 TS=(non-invasive brain stimulation OR noninvasive brain stimulation OR transcranial electrical stimulation OR transcranial direct current stimulation OR transcranial alternating current stimulation OR transcranial randomnoise stimulation OR transcranial magnetic stimulation OR repetitive transcranial magnetic stimulation OR theta burst stimulation OR TES OR tDCS OR tACS OR tRNS OR TMS OR rTMS OR TBS) 47825

#3 TS=(sleep* OR sleep disorder* OR sleep wake disorder OR sleep-related neurogenic Tachypnea* OR Sleeper Syndrome* OR dyssomnia* OR Nocturnal Eating drinking Syndrome* OR Sleep Initiation and Maintenance disorders OR Insomnia OR disorders of Initiating and Maintaining Sleep OR dIMS OR Sleeplessness OR Early Awakening OR parasomnia* OR hypersomnia OR Circadian OR restless leg syndrome OR periodic limb OR polysomnography OR narcolepsy OR somnolence) 237656

#4 #1 AND #2 AND #3 41

7.Database: **Embase (Embase.com)**

Date: 2023-11-22

Number of results:152

Search strategy:

('stroke'/exp OR 'stroke*':ab,ti OR 'apoplexy':ab,ti OR 'cerebrovascular':ab,ti OR 'hemiplegia':ab,ti OR 'cerebral hemorrhage':ab,ti OR 'cerebral infarction':ab,ti OR 'cerebral stroke*':ab,ti OR 'cerebral vascular accident*':ab,ti OR 'brain vascular accident*':ab,ti OR 'cva':ab,ti) AND ('transcranial magnetic stimulation'/exp OR 'transcranial direct current stimulation'/exp OR 'non-invasive brain stimulation':ab,ti OR 'noninvasive brain stimulation':ab,ti OR 'transcranial electrical stimulation':ab,ti OR 'transcranial direct current stimulation':ab,ti OR 'transcranial alternating current stimulation':ab,ti OR 'transcranial randomnoise stimulation':ab,ti OR 'transcranial magnetic stimulation':ab,ti OR 'repetitive transcranial magnetic stimulation':ab,ti OR 'theta burst stimulation':ab,ti OR 'tes':ab,ti OR 'tdcs':ab,ti OR 'tacs':ab,ti OR 'trns':ab,ti OR 'tms':ab,ti OR 'rtms':ab,ti OR 'tbs':ab,ti) AND ('sleep'/exp OR 'sleep wake disorders'/exp OR 'sleep*':ab,ti OR 'sleep disorder*':ab,ti OR 'sleep wake disorder':ab,ti OR 'sleep-related neurogenic tachypnea*':ab,ti OR 'sleeper syndrome*':ab,ti OR 'dyssomnia*':ab,ti OR 'nocturnal eating drinking syndrome*':ab,ti OR 'sleep initiation and maintenance disorders':ab,ti OR 'insomnia':ab,ti OR 'disorders of initiating and maintaining sleep':ab,ti OR 'dims':ab,ti OR 'sleeplessness':ab,ti OR 'early awakening':ab,ti OR 'parasomnia*':ab,ti OR 'hypersomnia':ab,ti OR 'circadian':ab,ti OR 'restless leg syndrome':ab,ti OR 'periodic limb':ab,ti OR 'polysomnography':ab,ti OR 'narcolepsy':ab,ti OR 'somnolence':ab,ti)

8.Database: **Cochrane Library (cochranelibrary.com)**

Date: 2023-11-22

Number of results: 181 records for 179 trials

Search strategy:

#1 MeSH descriptor: [Stroke] explode all trees 15230

#2 (stroke* or apoplexy or cerebrovascular or hemiplegia or cerebral hemorrhage or cerebral infarction or cerebral stroke* or cerebral vascular accident* or brain vascular accident* or CVA):ti,ab,kw (Word variations have been searched) 84858

#3 #1 OR #2 84971

：cerebrovascular disorders、stroke、cerebrovascular event、

poststroke、apoplex y、cerebrovascular、accident、brainvascular accident、CVD、

cerebral/brain infarction、brain ischemia、intracranial embolism、cerebral/brain h（a）emorrhage、intracranial h（a）emorrhage；

#4 MeSH descriptor: [Transcranial Magnetic Stimulation] explode all trees 2359

#5 MeSH descriptor: [Transcranial Direct Current Stimulation] explode all trees 1787

#6 (non-invasive brain stimulation or noninvasive brain stimulation or transcranial electrical stimulation or transcranial direct current stimulation or transcranial alternating current stimulation or transcranial randomnoise stimulation or transcranial magnetic stimulation or repetitive transcranial magnetic stimulation or theta burst stimulation or TES or tDCS or tACS or tRNS or TMS or rTMS or TBS):ti,ab,kw (Word variations have been searched) 1787

#7 #4 or #5 or #6 19312

#8 MeSH descriptor: [Sleep] explode all trees 9588

#9 MeSH descriptor: [Sleep Wake Disorders] explode all trees 11181

#10 (sleep* or sleep disorder* or sleep wake disorder or sleep-related neurogenic Tachypnea* or Sleeper Syndrome* or dyssomnia* or Nocturnal Eating drinking Syndrome* or Sleep Initiation and Maintenance disorders or Insomnia or disorders of Initiating and Maintaining Sleep or dIMS or Sleeplessness or Early Awakening or parasomnia* or hypersomnia or Circadian or restless leg syndrome or periodic limb or polysomnography or narcolepsy or somnolence):ti,ab,kw (Word variations have been searched) 75032

#11 #8 or #9 or #10 75136

#12 #3 and #7 and #11 181

9.Database: **scopus**

Date: 2023-11-22

Number of results: 150

Search strategy:

(TITLE-ABS-KEY(“stroke*” OR “apoplexy” OR “cerebrovascular” OR “hemiplegia” OR “cerebral hemorrhage” OR “cerebral infarction” OR “cerebral stroke*” OR “cerebral vascular accident*” OR “brain vascular accident*” OR “CVA”)) AND (TITLE-ABS-KEY(“non-invasive brain stimulation” OR “noninvasive brain stimulation” OR “transcranial electrical stimulation” OR “transcranial direct current stimulation” OR “transcranial alternating current stimulation” OR “transcranial randomnoise stimulation” OR “transcranial magnetic stimulation” OR “repetitive transcranial magnetic stimulation” OR “theta burst stimulation” OR “TES” OR “tDCS” OR “tACS” OR “tRNS” OR “TMS” OR “rTMS” OR “TBS”)) AND (TITLE-ABS-KEY(“sleep*” OR “sleep disorder*” OR “sleep wake disorder” OR “sleep-related neurogenic Tachypnea*” OR “Sleeper Syndrome*” OR “dyssomnia*” OR “Nocturnal Eating drinking Syndrome*” OR “Sleep Initiation and Maintenance disorders” OR “Insomnia” OR “disorders of Initiating and Maintaining Sleep” OR “dIMS” OR “Sleeplessness” OR “Early Awakening” OR “parasomnia*” OR “hypersomnia” OR “Circadian” OR “restless leg syndrome” OR “periodic limb” OR “polysomnography” OR “narcolepsy” OR “somnolence”))

# Supplementary Figures and Tables

For more information on Supplementary Material and for details on the different file types accepted, please see [here](https://www.frontiersin.org/guidelines/author-guidelines#supplementary-material).

## Supplementary Tables

| **Supplementary Table 1 Basic characteristics of included studies** | | | | | | | | | | | | | | |
| --- | --- | --- | --- | --- | --- | --- | --- | --- | --- | --- | --- | --- | --- | --- |
| Study | Sample size(E/C) | Age (E/C, years) | type of stroke | Patients | Diagnostic criteria | | Interventions | | Stimulation frequency (Hz) | stimulated areas | stimulated coil | Stimulus intensity | Duration | Outcome indicators |
|  |  |  |  |  | Stroke | Sleep disorder | C | E |  |  |  |  |  |  |
| Luo^[1]^,2016 | 55/55 | 62.73±6.07/61.24±5.42 | Ischemic and hemorrhagic stroke | PSI | ②① | ⑥ | alprazolam tablet | rTMS | 1Hz | − | − | 500Gs | 30min/d, 4wks | PSQI、Adverse reactions |
| Chen^[2]^,2018 | 32/32 | 64.2±6.9/66.5±6.9 | Ischemic and hemorrhagic stroke | PSI | ⑥① | ⑤ | Alprazolam | rTMS | 1Hz | FC | − | 500Gs | 30min/d, 6d/wk, 4wks | PSQI |
| Sheng^[3]^,2019 | 50/48 | 61.17±12.56/57.24±11.1 | Ischemic and hemorrhagic stroke | PSSD | ②① | ② | Dexzopiclone Tablets | rTMS | 1mHz20min after 11mHz5min | PCZ | − | 500Gs | 20min/d, 5d/wk, 2wks | PSQI、PSG |
| Zhu^[4]^,2019 | 30/30 | 65.97±10.51/65.90±9.50 | ischemic stroke | PSI | ②① | ⑤② | Zolpidem Tartrate Tablets＋false stimulus | Zolpidem Tartrate Tablets＋ILF-TMS | 0.2Hz | − | Circular coil | 500GS | 20min/d, 10ds | PSQI、PSG |
| Chen^[5]^,2020 | 32/31 | 64.06±6.82/65.16±9.18 | Ischemic and hemorrhagic stroke | PSI | ②① | ②⑤ | Escitalopram +false stimulus | Escitalopram +rTMS | 10Hz | l-DLPFC | Figure-of-eight coil | 90%MT | 1session/d, 10 consecutive treatment days. 4wks | PSQI、PSG、HAMD-17 |
| Ding^[6]^,2020 | 46/46 | 70±4/72±4 | Ischemic and hemorrhagic stroke | PSSD | ②① | ② | false stimulus | rTMS | 1Hz | b-DLFC | − | 80%MT | 1session/d,, 2wks | PSQI、PSG、BDNF、Adverse reactions |
| Armalia^[7]^,2021 | 24/24 | 18-65 | Ischemic stroke | PSSD | ① | ② | Medication (not specified) | Medication (not specified)＋rTMS | − | − | − | − | − | PSQI |
| Chen^[8]^,2021 | 45/45 | 70±5/70±5 | Ischemic and hemorrhagic stroke | PSSD | ④① | ① | Bailemian capsules＋false stimulus | Bailemian capsules＋rTMS | 10Hz | b-DLFC | Figure-of-eight coil | 80%RMT | 1session/d, 5ds/wk, 4wks | PSQI、BDNF、Adverse reactions |
| Xu^[9]^,2021 | 30/28 | 65.7±6.1/64.2±5.9 | Ischemic and hemorrhagic stroke | PSI | ②① | ⑩ | alprazolam tablets | rTMS | 1Hz | r-DLFC | Figure-of-eight coil | 500Gs | 20min/d, 1mo | PSQI、Adverse reactions |
| Dong^[10]^,2022 | 43/43 | 60.96±5.13/61.32±4.67 | Ischemic and hemorrhagic stroke | PSI | ④ | ① | Escitalopram oxalate tablets＋false stimulus | Escitalopram oxalate tablets＋rTMS | 10Hz | − | Figure-of-eight coil | 80%MT | 20min/d, 5ds/wk, 4wks | PSQI、HAMD-17、BDNF |
| Gu^[11]^,2022 | 22/22 | 54.2±12.66/58.6±12.58 | Ischemic and hemorrhagic stroke | PSI | ②① | ③ | false stimulus | tDCS | 2mA | Anode l-DLPFC  Cathode r-DLPFC | Bipolar Electrode Pads | 2mA | 20min/d, 5ds/wk, 4wks | PSQI、PSG、HAMD-17 |
| Han^[12]^,2022 | 44/43 | 53.68±7.52/55.23±7.79 | Ischemic and hemorrhagic stroke | PSSD | ① | ①② | Fluoxetine Hydrochloride Dispersible Tablets | Fluoxetine Hydrochloride Dispersible Tablets＋HD-tDCS | 2mA | l-DLPEC | High definition circular electrode (Diameter < 12 mm) | − | 1session/d, 4wks | PSQI、HAMD-17 |
| Huang^[13]^,2022 | 45/45 | 61.06±4.65/61.20±4.69 | Ischemic stroke | PSI | ④ | ② | Dexzopiclone tablets | rTMS | 1Hz | r-DLPFC | Figure-of-eight coil | − | 20min/d, 14ds | PSQI |
| Qi^[14]^,2022 | 46/45 | 63.12±6.07/63.75±5.92 | Ischemic and hemorrhagic stroke | PSI | ⑦① | ③ | Alprazolam | rTMS | 1Hz | r-DLFC | Figure-of-eight coil | 80%~120%MT | 20min/d, rest 2ds after 5ds of treatment, 1mo | PSQI、Adverse reactions |
| Xiao^[15]^,2022 | 30/30 | − | Ischemic stroke | PSSD | ⑤ | ⑦② | Estazolam +false stimulus | Estazolam +rTMS | 1Hz | b-DLPFC and POR | Figure-of-eight coil | 120%MT | 30min/d, 2wks | PSG、BDNF、Adverse reactions |
| Zhang^[16]^,2022(A) | 13/13 | 59.33±8.25/59.50±11.18 | Ischemic and hemorrhagic stroke | PSSD | ②① | ④ | Routine rehabilitation | Routine rehabilitation＋TBS | 3 pulses/clump, inter-clump: 5Hz, intra-clump: 50Hz | r-DLPFC | Figure-of-eight coil | 70%RMT | 1session/d, 7ds/wk, 2wks | PSQI、HAMD-17 |
| Zhang^[16]^,2022(B) | 13/13 | 63.75±9.11/59.50±11.18 | Ischemic and hemorrhagic stroke | PSSD | ②① | ④ | Routine rehabilitation | Routine rehabilitation＋rTMS | 1Hz | r-DLPFC | Figure-of-eight coil | 80%RMT | 25min/d, 7ds/wk, 2wks | PSQI、HAMD-17、Adverse reactions |
| Zhong^[17]^,2022 | 50/50 | 63.86±8.78/62.88±7.99 | Ischemic stroke | PSI | ③① | ⑨ | Routine rehabilitation | Routine rehabilitation＋rTMS | 1Hz | − | Circular coil | − | 20min/d, 7ds | PSQI |
| An^[18]^,2023 | 42/45 | 60±8/61±8 | Ischemic stroke | PSI | ③ | ⑧ | Estazolam | rTMS | 1Hz | r-DLFC | Figure-of-eight coil | 80%~120%MT | 1session/d, 4wks | PSQI、PSG、Adverse reactions |
| Stroke Diagnostic Criteria:  Stroke diagnostic criteria include: ① confirmation via magnetic resonance imaging (MRI) or computed tomography (CT); ② criteria as defined by the Fourth National Academic Conference on Cerebrovascular Disease; ③ diagnostic guidelines from the "China Acute Ischemic Stroke Diagnostic and Treatment Guidelines, 2018"; ④ criteria from the "China Cerebrovascular Disease Prevention and Treatment Guidelines"; ⑤ criteria outlined in the "Various Types of Cerebrovascular Disease" by the Chinese Medical Association; and ⑥ standards set by the Neurology Committee of the Chinese Society of Traditional Chinese Medicine in 2006. ⑦ diagnoses that align with the combined Chinese and Western medicine criteria for cerebral infarction and cerebral hemorrhage, as formulated by the Neurology Committee of the Chinese Society of Integrative Medicine in 2006.  Diagnostic Criteria for Sleep Disorders:  Sleep disorders are diagnosed based on: ① polysomnography indicating a disorder; ② the Pittsburgh Sleep Quality Index (PSQI); ③ criteria from the "Chinese Adult Insomnia Diagnostic and Treatment Guidelines, 2017 Edition"; ④ criteria in the International Classification of Sleep Disorders, Third Edition (ICSD-3); ⑤ criteria from the third revised edition of the Chinese Classification and Criteria for the Diagnosis of Mental Disorders (CCMD-3-R); ⑥ earlier criteria in the second revised edition (CCMD-2-R); ⑦ the Diagnostic and Statistical Manual of Mental Disorders, Fifth Edition (DSM-V) from the United States; ⑧ the 2012 "Chinese Guidelines for the Diagnosis and Treatment of Adult Insomnia"; ⑨ general insomnia diagnostic criteria covering difficulty in falling asleep, sleep maintenance issues, early awakening, degraded sleep quality, or non-restorative sleep; ⑩ criteria by the Revised Sleep Disorders Group of the Neurology Branch of the Chinese Medical Association.  Stimulation Sites and Stimulus Intensity:  Stimulation sites are denoted as follows: DLPFC (dorsolateral prefrontal cortex), b-DLPFC (bilateral dorsolateral prefrontal cortex), l-DLPFC (left dorsolateral prefrontal cortex), r-DLPFC (right dorsolateral prefrontal cortex), POR (parietal-occipital region), and r-DLFC (right dorsolateral frontal cortex). The frontal cortex is abbreviated as FC, and b-DLFC represents the bilateral dorsolateral frontal cortex. PCZ is defined as 1 cm posterior to the parietal CZ area.  Stimulus intensity is indicated by MT (motor threshold), RMT (resting motor threshold), and GS (Gauss). | | | | | | | | | | | | | | |

## Supplementary Figures


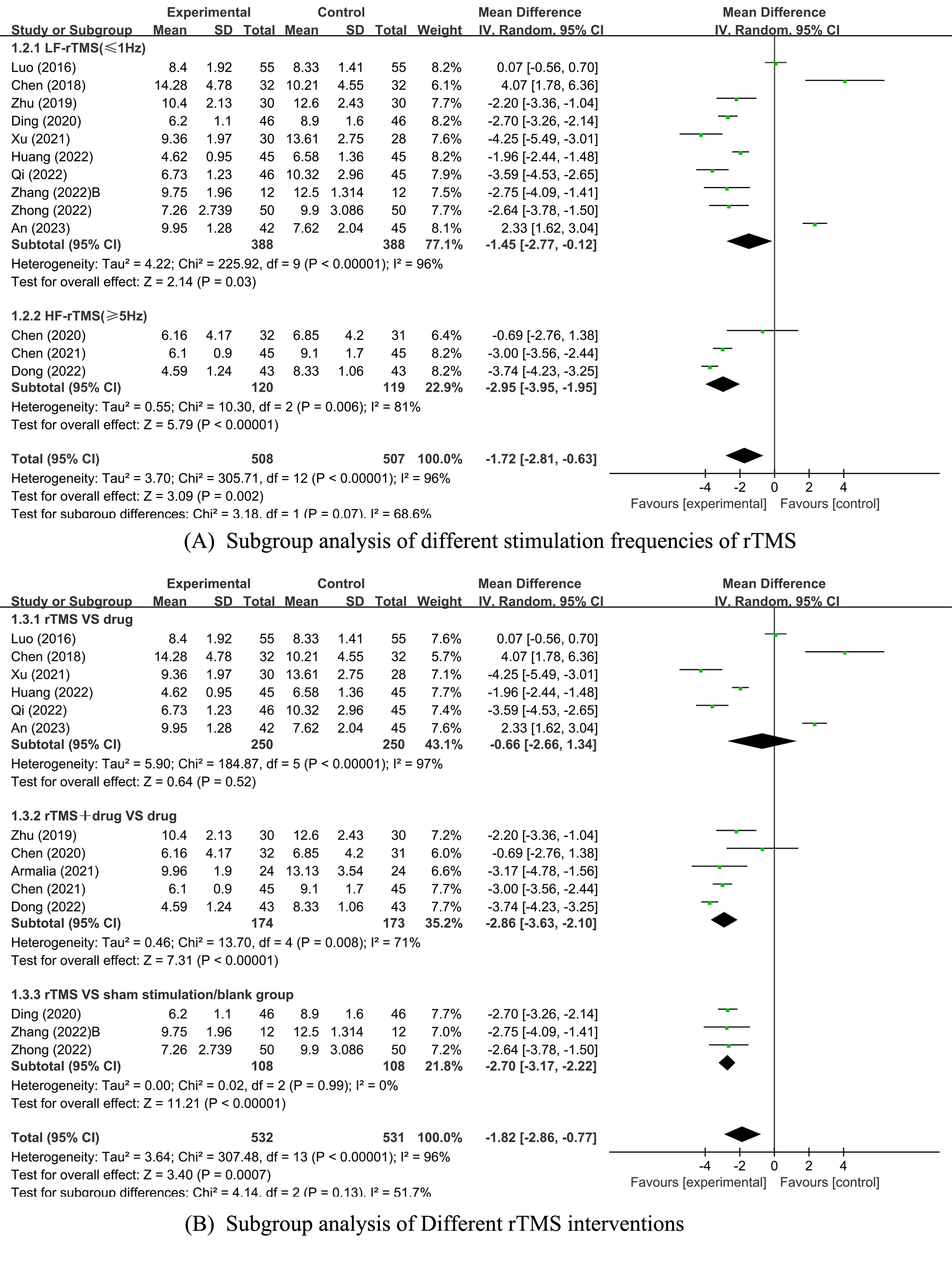


Supplementary Figure 1　Subgroup analysis of the effect of rTMS on PQSI scores in patients with PSSD


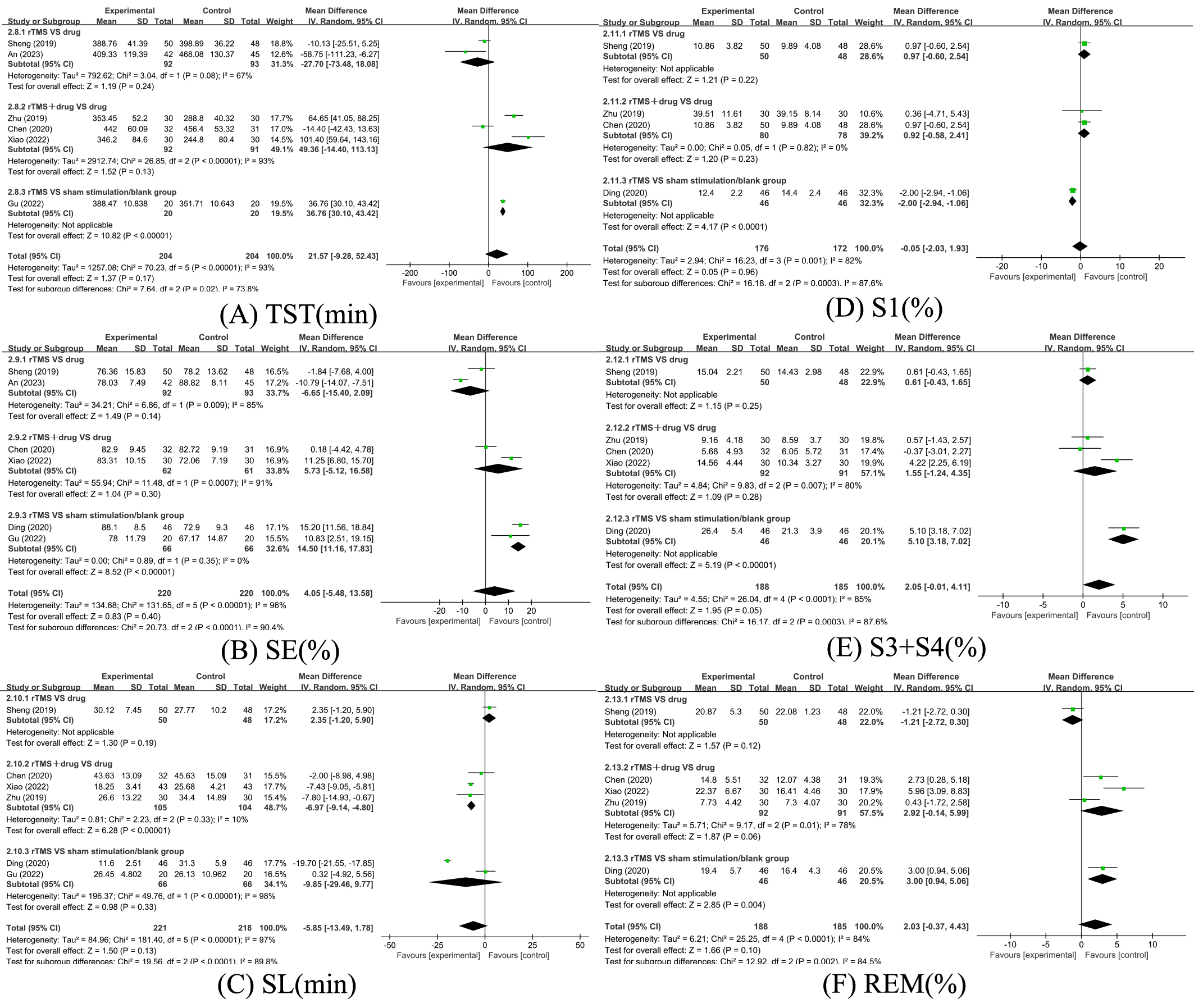


Supplementary Figure2　Subgroup analysis of the effect of rTMS on PSG sleep parameters in PSSD patients

**
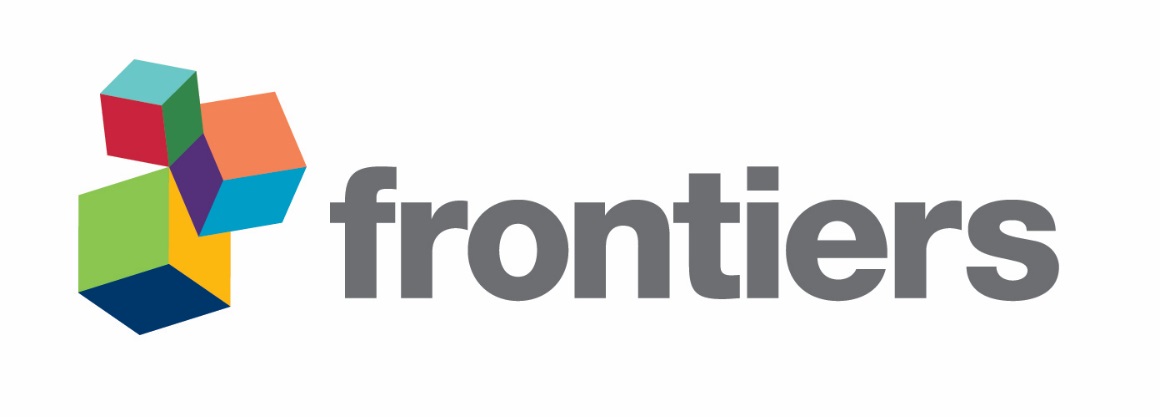
**

**Supplementary Figure 1.** The figure legends are required to have the same font as the main text, 12 point normal Times New Roman, single spaced. Please use a single paragraph for each legend and prepare the figures keeping in mind the PDF layout.

[1] Luo H, Wang C, Gu J. Curative observation of transcranial magnetic stimulation in insomnia in patients after stroke[J]. Chin J Neuromed, 2016, 15(4): 403-405.

[2] Chen P, Chen Z, Li M, et al. Clinical study of repetitive transcranial magnetic stimulation for insomnia after stroke[J]. Journal of Hubei University of Medicine, 2018, 37(03): 262-264.

[3] Sheng W, Yang X, Lyu L, et al. Efficacy of TMS Combined with Music Electro-Acupuncture in Treating Post-Stroke Sleep Disorders: A Clinical Observation by Polysomnography[J]. Journal of Clinical Acupuncture and Moxibustion, 2019, 35(04): 7-12.

[4] Zhu M, Liu Y, Huang J, et al. Clinical study on infra-low frequency transcranial magnetic stimulation in the treatment of insomnia after ischemic stroke [J]. Journal of Neuroscience and Mental Health, 2019, 19(5): 462-466.

[5] Chen L, Fu S, Gu Z, et al. The clinical study of high frequency repetitive transcranial magnetic stimulation ( rTMS) on the

treatment of poststroke depression with insomnia[J]. Guangzhou Medical Journal, 2020, 51(06): 28-32.

[6] Ding L, Xiao B, Chen X, et al. Effects of repetitive transcranial magnetic stimulation on sleep structure and brain-derived neurotrophic factors in patients with post-stroke sleep disorders[J]. Journal of Practical Medical Techniques, 2020, 27(02): 153-156.

[7] Armalia, Tammasse J, Akbar M, et al. The influence of repetitive transcranial magnetic stimulation toward improvement of post ischemic stroke patient's quality of sleep[J]. Medicina Clinica Practica, 2021, 4.

[8] Chen L, Fu Q. Effects of high-frequency repetitive transcranial magnetic stimulation in the treatment of post-stroke sleep disorders and its effect on patients' sleep quality serum brain-derived neurotrophic factor levels[J]. Shanxi Med J, 2021, 50(06): 981-984.

[9] Xu D, Tian J, Chen C, et al. Effect of low-frequency repetitive transcranial magnetic stimulation on insomnia after stroke[J]. Chin J Neuroimmunol &Neuro, 2021, 28(01): 58-60+66.

[10] Dong X. Efficacy of high-frequency transcranial magnetic stimulation in the treatment of post-stroke depression with insomnia[J]. Medcine And Health, 2022(6).

[11] Gu B, Huang F, Li H, et al. Effect of transcranial direct current stimulation on post-stroke insomnia[J]. Chin J Rehabil Theory Pract, 2022, 28(12): 1466-1472.

[12] Han L. Effect of high definition transcranial direct current stimulation combined with Fluoxetine Hydrochloride in the treatment of post -stroke patients with depression with sleep disorders[J]. China Medical Herald, 2022, 19(29): 94-96+100.

[13] Huang D. To Observe the Clinical Effect of Ｒepeated Transcranial Magnetic Stimulation on Insomnia Patients with Ischemic Stroke[J]. World Journal of Sleep Medicine, 2022, 9(02): 209-211.

[14] Qi W, Qi G, Liang Y, et al. Effect of repetitive transcranial magnetic stimulation on post-stroke insomnia, anxiety, and depression[J]. China Modern Doctor, 2022, 60(12): 100-103.

[15] Xiao Y, Yan L, Chen B. Eff ect of repeated transcranial magnetic stimulation combined with estazolam in the treatment of sleep disorders after ischemic stroke[J]. Journal of Changchun University of Chinese Medicine, 2022, 38(08): 906-909.

[16] Zhang Z. A comparative study on the efficacy of low-frequency repetitive transcranial magnetic stimulation in routine mode and burst mode on post-stroke sleep disorders[D]. Liaoning: China Medical University, 2022.

[17] Zhong Z, Li Y, Luo S, et al. Observation of low-frequency repetitive transcranial magnetic stimulation in the treatment of insomnia after acute cerebral infarction[J]. China Prac Med, 2022, 17(11): 1-4.

[18] An F, Liu J, Gu L, et al. Effect of balancing acupuncture plus low-frequency rTMS on sleep quality in post-stroke insomnia patients[J]. Shanghai J Acu-mox, 2023, 42(08): 784-790.
